# Evaluating employees’ positive functioning at work: Adaptation and validation of the PERMA+4 scales in the Italian context

**DOI:** 10.1371/journal.pone.0355712

**Published:** 2026-08-13

**Authors:** Alessandro Concas, Kristina Shea, Irene Garigali, Andrea Bonanomi, Daniel J. Martin, Stewart I. Donaldson

**Affiliations:** 1 University of Cagliari, Cagliari, Italy; 2 Claremont Graduate University, Claremont, California, United States of America; 3 University of Bologna, Bologna, Italy; 4 Università Cattolica del Sacro Cuore, Milan, Italy; University of Botswana, BOTSWANA

## Abstract

As positive psychology continues to gain prominence in organizational contexts, scholars are calling for more cross-cultural investigations into how well-being frameworks translate across different cultural contexts. This study validated both the brief and extended versions of the PERMA+4 Positive Functioning at Work Scale in the Italian context using a sample of 673 employed participants. The PERMA+4 framework extends the original PERMA model, which includes positive emotions, engagement, relationships, meaning, and accomplishment, by adding four additional dimensions: physical health, mindset, environment and economic security. The scales demonstrated robust psychometric properties with excellent overall reliability. For the extended version, confirmatory factor analysis supported both the presence of a nine-factor correlated structure and a Positive Functioning at Work general factor, with the bifactor model showing the best fit. For the brief version, item response theory analyses indicated good item functioning, while confirmatory factor analysis confirmed the expected one-factor structure. Measurement invariance was tested and established for both versions across a broad range of sociodemographic variables. The study highlights that the two versions of the scale present high convergence and a consistent nomological pattern. Both scales demonstrated strong convergent validity through high correlations with work engagement, job satisfaction, life satisfaction, and burnout, and showed comparable associations with work performance outcomes. Moreover, the four additional PERMA+4 dimensions demonstrated incremental validity beyond the original PERMA ones. The Italian validation further supports PERMA+4’s cross-cultural applicability while revealing culturally specific patterns. This research provides both Italian organizations and academics with a comprehensive, evidence-based tool for assessing workplace well-being that moves beyond deficit-focused approaches toward positive functioning assessment.

## Introduction

Positive psychology (PP), the scientific study of well-being [1], emerged more than two decades ago in response to a recognized imbalance within the psychological sciences. Conventional approaches, which include those applied to organizational settings, have predominantly concentrated on deficits such as burnout, stress, and turnover. PP instead adopts a complementary perspective, treating organizations as dynamic systems and asking how optimal functioning can foster thriving and flourishing, thereby extending rather than replacing the insights gained from the study of dysfunction [2–4]. While workplace well-being has received growing attention, positive psychology has been criticized for its Western-centric assumptions about well-being, prompting calls for greater attention to cultural variation in how well-being is conceptualized and measured, as measurement non-invariance across contexts can limit the validity of cross-cultural comparisons [5,6].

### Theoretical background

One of the most influential constructs to emerge from positive psychology is PERMA, a framework that articulates five major building blocks contributing to well-being: Positive Emotions, Engagement, Relationships, Meaning, and Accomplishment [7]. The PERMA framework has been adapted and examined within the workplace using the Workplace PERMA Profiler [8] to analyze workplace well-being. Donaldson and Donaldson [9] have since built on PERMA by identifying four additional contextual building blocks, going beyond the role of merely psychological factors in determining workplace well-being, thereby proposing the PERMA+4 framework. The following is a brief exploration of each of the building blocks and their conceptualization from Donaldson and Dubin [10]:

1) *Positive emotions* – In the here and now, experiencing happiness, gratitude, optimism, joy, love and other emotions that bring positive connection and positive functioning.
2) *Engagement* – Experiencing flow or being highly absorbed in the activities of your life or in the workplace.
3) *Relationships* – Experiencing high quality connections and mutually beneficial relationships with those around you which provide support, care, comfort, connection, love and appreciation.
4) *Meaning* – Being connected to something larger than yourself in life, and serving a higher calling, purpose, and meaning in life.
5) *Accomplishment* – Achieving challenging life goals and having a specific sense of mastery for a topic, area, or domain of interest.
6) *Physical health* – High levels of positive biological, functional, psychological, and physiological health.
7) *Mindset* – Striving for a growth mindset in which optimism, hope, and having a future-oriented view of life helps us see opportunities as growth and setbacks as learning opportunities.
8) *Environment* – Assessing the quality of the physical environment which includes spatiotemporal elements such as access to natural light, physical safety, and other environmental components.
9) *Economic security* – Having financial security, and stability and meeting individual and collective financial needs.

Initial research studies established PERMA+4 as a workplace well-being framework, validating the long and the short version of the Positive Functioning at Work Scale [9,11], built to measure its nine dimensions in the workplace. Moreover, the studies examined its role as a predictor of positive work outcomes, such as reduced turnover intentions and higher levels of adaptivity, proactivity, and proficiency using both self and coworker reports [9,12]. Subsequent research has strengthened these findings: longitudinal evidence confirmed that PERMA+4 dimensions predict work-related outcomes over time [13], and a systematic review found that PERMA+4 was a stronger predictor of subjective well-being than PERMA alone [14]. While these findings provide support for the PERMA+4 as a measurement framework, further research is needed to examine its dimensions across diverse cultural and economic contexts.

### Cross-cultural validity

Numerous validation studies have verified the factorial structure of the Positive Functioning at Work Scale across diverse cultural settings. For instance, consistent evidence of adequate to good model fit was reported for the multidimensional models of the long version of the scale (best model fit range reported among individual studies: comparative fit index [CFI] =.91–.98; root mean square error of approximation [RMSEA] =.04–.06), both in the original United States study (n = 727; [9]), a subsequent US evaluation (n = 406; [15]), as well as in Spain (n = 698; [16]), Germany (n = 379; [17]), and Turkey (n = 297; [18]). Notably, these investigations employed various multidimensional modeling strategies (e.g., bifactor [9,16] and hierarchical structures [8, 15, 17, 18]) yet converged in supporting both the nine-dimensional configuration and the existence of a general factor of positive functioning at work. This conclusion is further corroborated by an international psychometric assessment (n = 1200; [12]) and the validation of short forms in Russian (n = 325; [19]) and Turkish (n = 297 [18]) populations (best model fit range reported among the individual studies: CFI = 0.97–0.99; RMSEA = 0.05–0.08), which extended the evidence to the 9-item brief scale, underscoring both the existence of multidimensionality within the factorial structure and the appropriateness of a global indicator of positive functioning at work.

Total scale reliability in literature has been reported to be high for both the long (ω = .94–.98) and short form (ω = .88–.91). At the subscale level, positive emotions (ω = .85–.93) and meaning (ω = .85–.91) are consistently the strongest, while economic security and environment the weakest, particularly in the Turkish sample (ω = .63 and .62). This behavior, while occasional in economic security, tends to be systematic in the environment dimension [9,15–18]. Moreover, while positive emotions, meaning and mindset tend to consistently have among the highest factor loadings and measurement precision across the validation, economic security tends to be problematic, showing the weakest contribution to the general factor likely because, unlike other contextual dimensions, it is particularly shaped by macro-level conditions beyond both individual and organizational control [18,19].

Beyond structural validity, these studies also provide evidence of external validity, showing cross-culturally consistent associations with general well-being and significant predictive relationships with key organizational outcomes [9,15–19].

Finally, the literature provided evidence of measurement invariance across work role [9] and educational level [16], whereas only configural invariance has been supported for gender and metric invariance for ethnicity [15]. However, existing evidence for measurement invariance is relatively limited, derives from single-context studies and has not been further replicated.

### The Italian context

Although the preliminary cross-cultural validation studies of PERMA+4 show promising results, no study has yet examined this framework in the Italian context. Doing so is warranted on both substantive and methodological grounds. The Italian labor market presents several structural features that distinctively shape workers’ well-being. Widespread skills mismatch, particularly among younger workers, reduces job satisfaction and increases vulnerability to disengagement and burnout [20–22]. These challenges are compounded by high levels of precarity and comparatively lower salaries, which fuel economic insecurity and negatively affect mental health [23–25], as well as acute territorial disparities between Southern and Northern regions in terms of both employment opportunities and perceived well-being [26,27].

A further distinctive element is the predominance of small and micro enterprises (SMEs) in the Italian economic fabric. These organizations face significant financial, organizational, and contextual constraints that limit their capacity to address workplace well-being systematically [28,29]. Moreover, well-being in SMEs appears to rely on partly different mechanisms, and interventions developed for larger organizations often do not yield comparable results when applied to smaller settings [30], underscoring the need for approaches tailored to their specific constraints and organizational realities [31]. These distinctive traits of the Italian work landscape underscore the importance of examining how instruments originally developed in other contexts function when transferred.

Moreover, Italy has a well-established tradition of positive organizational scholarship and healthy organization research, which emphasizes flourishing, resilience, and the interconnection between individual and organizational well-being through a strengths-based rather than deficit-focused approach [32–34]. This stream has produced several context-specific measures and validated well-being instruments, reflecting sustained interest in positive functioning in the Italian context [35–37]. However, this body of work remains largely construct-specific and does not yet provide a comprehensive multidimensional framework integrating psychological and contextual determinants of workplace well-being for the Italian workforce, which continues to rely substantially on deficit-oriented assessment approaches [38,39].

PERMA+4 fills this gap by combining the five psychological components of PERMA with four contextual dimensions, physical health, mindset, environment, and economic security which creates a structure well-suited for Italy, where economic insecurity and environmental and organizational constraints shape workers’ experiences. Validating PERMA+4 in Italy helps identify protective factors to support well-being and inform tailored interventions.

### The present study

In this study, we seek to address the call for cross-cultural validation by adapting the PERMA+4 Positive Functioning at Work Scale within an Italian context. Specifically, both the 29-item and 9-item versions of the scale are validated and compared, assessing factorial structure through confirmatory factor analysis and examining reliability, convergent, discriminant and criterion validity, as well as the degree of convergence between the two forms. Item response theory is additionally applied to the short form to further examine item functioning. Finally, measurement invariance is tested among different relevant sociodemographic and work variables.

## Methods

This study has received exempt approval from the Institutional Review Board at the Claremont Graduate University (Protocol # 5136). Ethical considerations for this study included voluntary participation, confidentiality, and anonymity.

### Translation of PERMA+4 scales

The Italian version of the PERMA+4 Positive Functioning at Work Scale was developed following established international guidelines for cross-cultural adaptation of psychological instruments [40]. The translation process employed a rigorous forward-backward translation methodology involving independent translators with native-level proficiency in both languages. Two forward translations were conducted: one by a psychology expert to ensure conceptual equivalence and another providing colloquial translation to minimize professional bias. These were synthesized through expert consensus, followed by two independent backward translations prioritizing conceptual rather than literal equivalence. A final harmonization phase resolved ambiguities through documented consensus-building, with external review ensuring optimal cultural adaptation while preserving the original instrument’s psychometric properties [40,41]. S1 and S2 Tables in the supplementary materials include both the original English questionnaires and the Italian translations.

### Participants

Data were collected between July 10^th^, 2025 and July 16^th^, 2025, through the Qualtrics web-based platform using a community sample from Italy. Participants were recruited through Prolific, an online platform that ensures high-quality paid samples for research purposes [42] and paid an hourly wage of $11.87 for their participation in the study. All participants provided written informed consent before participating in the study, ensuring that they understood the study’s purpose, procedures, and potential risks before agreeing to participate. Participation was voluntary and could be discontinued at any time. Participants were Italian workers aged 18 or older, currently employed in any sector. Individuals who were unemployed or not actively working were excluded from participation. Initially, a data screening and cleaning process was conducted. The data did not have any missing values due to mandatory response requirements, though participants were provided with “Other” or “I prefer not to answer” options for unclear or sensitive questions. Response times were monitored to ensure data quality.

The questionnaire was structured to minimize response burden by incorporating cognitive breaks and attention checks. The scales under validation were administered first, followed by criterion validity measures. Convergent and discriminant validity instruments were presented subsequently in randomized order to balance response quality across participants. Socio-demographic information was collected at the end of the survey.

Men represented 53.3% of the sample, while women comprised 44.7%, with the remaining 2% identifying as non-binary or preferring not to disclose. Most participants (82.5%) fell within the 25–54 age range, and the sample demonstrated a high educational level, with 69.1% holding at least a bachelor’s degree. Geographically, participant distribution was relatively balanced across Italy’s regions, including North, Center, South, and Islands, closely mirroring the national population distribution [43]. Regarding employment characteristics, most participants were full-time (66.4%), standard employees (65.3%), and predominantly in white-collar positions (55.9%). Salary distribution aligned with national averages, with 49.8% earning between 1,000–2,000 euros monthly [43–44]. Work arrangements varied, with 48.9% working exclusively in-person and 65.5% reporting high levels of work autonomy. Work sectors, organizational tenure, and company sizes were heterogeneously distributed. Table 1 presents the participant demographics, which are broadly representative of the general population.

**Table 1:** Sociodemographic characteristics of the sample (N = 673).

Variable	Category	N	%
Gender	Female	301	44.73
	Male	359	53.34
	Non-binary	9	1.34
	Prefers not to answer	4	0.59
Age Group	18–24 years	54	8.02
	25–39 years	411	61.07
	40–54 years	144	21.40
	55–64 years	58	8.62
	65+ years	6	0.89
Education	Lower secondary diploma	13	1.93
	Upper secondary diploma	195	28.97
	Bachelor’s degree	166	24.67
	Master’s degree or higher	299	44.43
Employment	Full-time worker	447	66.42
	Part-time worker	149	22.14
	Student-worker	77	11.44
Monthly Income	Less than €1000	162	24.07
	€1000–1500	161	23.92
	€1501–2000	174	25.85
	€2001–2500	81	12.04
	More than €2500	49	7.28
	Prefers not to answer	46	6.84

Notes: N = absolute frequency; % = percentage of total sample. Income categories reflect monthly net income in euros. Upper secondary diploma includes post-secondary vocational qualification. Employment categories are mutually exclusive; participants working while enrolled in formal education were classified as student-workers regardless of hours worked. Full-time worker refers to participants working at least 35 hours per week

### Measures

*Positive Functioning at Work Scale – 29 item version (PFW-29) –* The PFW-29 scale, translated and adapted to the Italian context through a process of forward translation, back translation, and harmonization of results, is designed to measure positive functioning at work. The tool, developed by Donaldson and Donaldson [9], consists of 29 items that measure the degree of agreement with statements related to the nine dimensions of the PERMA+4 framework (e.g., “Overall, I am enthusiastic about my work”). The scale includes three items for each of the 9 dimensions, with physical health and relationships having four, making up the 29 items. The degree of agreement was measured using a Likert scale from 1 (“Strongly disagree”) to 7 (“Strongly agree”). In line with the literature, in this study the reliability of the instrument showed excellent values both at the scale (ω = .93; ωh = .86) and the dimensions level (ω > .80), with the exception of the accomplishment dimension, which still showed acceptable values (ω = .77), and the environment dimension, which showed slightly problematic values (ω = .65). For the Italian translation of the PFW-29 and the item-level statistics see Table 2.

**Table 2:** Italian translation of the PFW-29 and the item-level statistics.

Item	Italian Translation of the Items	M	SD	Skew.	Kurt.	λ Gen.	λ Dim.
PE-1	Provo gioia durante una tipica giornata di lavoro	4.15	1.39	−0.45	−0.36	0.77	0.37
PE-2	Globalmente, sono entusiasta del mio lavoro	4.52	1.50	−0.53	−0.37	0.80	0.53
PE-3	Amo il mio lavoro	4.47	1.57	−0.42	−0.47	0.82	0.38
EN-1	Solitamente mi lascio assorbire mentre lavoro su qualcosa che sfida le mie capacità	5.17	1.36	−0.91	0.76	0.60	0.45
EN-2	Perdo la cognizione del tempo mentre faccio qualcosa che mi piace a lavoro	5.16	1.36	−0.84	0.61	0.45	0.83
EN-3	Quando lavoro su qualcosa che mi piace, dimentico quello che ho intorno	5.06	1.33	−0.71	0.28	0.34	0.69
REL-1	Posso contare sul supporto dei miei colleghi se ne ho bisogno	4.98	1.44	−0.60	−0.07	0.41	0.74
REL-2	Mi sento apprezzato/a dai miei colleghi/e	5.04	1.27	−0.59	0.42	0.52	0.63
REL-3	Mi fido dei miei colleghi/e	4.84	1.32	−0.42	−0.08	0.45	0.78
REL-4	I miei colleghi e/o le mie colleghe tirano fuori il meglio di me	4.39	1.26	−0.19	0.19	0.57	0.61
MEAN-1	Il mio lavoro è significativo	4.75	1.50	−0.55	−0.21	0.79	0.54
MEAN-2	Capisco cosa rende il mio lavoro significativo	4.86	1.46	−0.63	0.02	0.75	0.53
MEAN-3	Il lavoro che faccio ha un fine superiore	4.09	1.75	−0.17	−0.90	0.64	0.46
ACC-1	Fisso degli obiettivi che mi aiutano a raggiungere le mie aspirazioni lavorative	4.49	1.50	−0.47	−0.29	0.70	0.16
ACC-2	Solitamente raggiungo ciò che mi prefiggo nel mio lavoro	5.11	1.21	−0.72	0.68	0.60	0.80
ACC-3	Sono perlopiù soddisfatto/a della mia performance a lavoro	5.18	1.23	−0.84	0.88	0.59	0.37
PH-1	Di solito mi sento fisicamente in salute	5.08	1.33	−0.78	0.27	0.49	0.65
PH-2	Mi ammalo raramente	5.17	1.39	−0.74	0.12	0.30	0.61
PH-3	Di solito posso superare le fonti di stress fisico (e.g., insonnia, infortuni, problemi di vista etc.)	4.97	1.29	−0.58	−0.09	0.45	0.61
PH-4	Sento di avere il controllo della mia salute fisica	4.67	1.38	−0.50	−0.16	0.42	0.78
MIND-1	Credo di poter migliorare le mie capacità lavorative attraverso un forte impegno	5.16	1.22	−0.80	0.99	0.61	0.17
MIND-2	Credo che il mio lavoro mi permetterà di crescere in futuro	4.67	1.62	−0.57	−0.37	0.76	0.65
MIND-3	Ho un futuro brillante all’interno dell’organizzazione per cui lavoro	3.85	1.60	−0.10	−0.68	0.74	0.25
ENV-1	Lo spazio fisico in cui lavoro (i.e., ufficio) mi permette di concentrarmi	4.51	1.57	−0.48	−0.37	0.65	0.30
ENV-2	C’è molta luce naturale nel mio luogo di lavoro	4.84	1.65	−0.67	−0.29	0.36	0.69
ENV-3	Posso facilmente accedere a spazi verdi dal mio ambiente lavorativo (e.g., parchi, mare, montagne etc.)	3.69	1.88	0.09	−1.14	0.24	0.45
ES-1	Mi sento a mio agio coi miei attuali guadagni	3.84	1.58	−0.08	−0.83	0.46	0.48
ES-2	Potrei perdere diversi mesi di paga a causa di una malattia importante, e avere comunque la mia sicurezza economica	3.45	1.91	0.26	−1.11	0.25	0.86
ES-3	Nel caso di un’emergenza finanziaria, ho risparmi sufficienti	3.92	1.85	−0.06	−1.06	0.29	0.80

Notes. M = mean; SD = standard deviation; Skew = skewness; Kurt = kurtosis; λ Gen. = factor loading on the general factor of the Bifactor model; λ Dim. = factor loading on the specific dimension factor. PE = Positive Emotions; EN = Engagement; REL = Relationships; MEAN = Meaning; ACC = Accomplishment; PH = Physical Health; MIND = Mindset; ENV = Environment; ES = Economic Security. Numeric suffixes (e.g., PE-1, PE-2) denote the position of the individual items within each dimension of the scale. All factor loadings are significant for p < 0.001

*Positive Functioning at Work Scale – 9 item version (PFW-9) –* The short version of the PFW scale [11], originally developed through Item Response Theory (IRT), consists of nine items assessing the degree of agreement with each of the nine dimensions of the PERMA+4 framework (e.g., “I set clear goals and achieve them”). The instrument was translated and adapted to the Italian context through a forward translation, back-translation, and harmonization process, aiming to measure positive functioning at work. Responses were collected on an 11-point Likert scale ranging from 0 (“Strongly disagree”) to 10 (“Strongly agree”). The instrument demonstrated high reliability in the present study (ω = 0.91). For the Italian translation of the PFW-9 and the item-level statistics see Table 3.

**Table 3:** Italian translation of the PFW-9 and the item-level statistics.

Item	Italian Translation of the Item	M	SD	Ass.	Kurt.	λ
PE	Mi sono sentito positivo a lavoro	7.01	2.26	−0.67	0.05	0.87
EN	Sono stato profondamente assorto e interessato al mio lavoro	6.96	2.23	−0.59	−0.01	0.87
REL	Sono stato incoraggiante e supportivo verso gli altri	7.57	2.06	−0.65	0.53	0.65
MEAN	Ho sentito che il lavoro che facevo aveva valore e ne valeva la pena	6.84	2.56	−0.49	−0.39	0.88
ACC	Ho stabilito degli obiettivi chiari e li ho raggiunti	7.27	2.24	−0.65	0.32	0.79
PH	Mi sono sentito fisicamente sano e forte	7.11	2.37	−0.54	−0.21	0.61
MIND	Ho sentito di avere un futuro brillante all’interno dell’organizzazione per cui lavoro	5.53	2.77	−0.01	−0.89	0.75
ENV	Lo spazio fisico in cui lavoro (i.e., ufficio) mi ha permesso di concentrarmi	6.77	2.60	−0.45	−0.55	0.67
ES	Mi sono sentito/a a mio agio coi miei attuali guadagni	5.99	2.76	−0.16	−0.90	0.50

Notes. M = mean; SD = standard deviation; Skew = skewness; Kurt = kurtosis; λ = factor loading. PE = Positive Emotions; EN = Engagement; REL = Relationships; MEAN = Meaning; ACC = Accomplishment; PH = Physical Health; MIND = Mindset; ENV = Environment; ES = Economic Security. All factor loadings are significant for p < 0.001

*Individual Work Performance Questionnaire (IWPQ-17) –* The Individual Work Performance Questionnaire (IWPQ-17) is a questionnaire developed by Koopmans et al. [45] and adapted to the Italian context and language by Platania et al. [46]. The tool consists of 17 items and three dimensions: task performance (TP), contextual performance (CP), and counterproductive work behaviors (CWB). The scale refers to the degree of agreement with certain statements regarding how the individual has performed their work over the last three months. The level of agreement is measured using a Likert scale from 1 (“Completely disagree”) to 5 (“Completely agree”). In line with the literature, the tool showed an acceptable-to-high level of reliability in this study (ω TP = 0.88; ω CP = 0.85; ω CWB = 0.73).

*Utrecht Work Engagement Scale – (UWES-9) –* The Utrecht Work Engagement Scale is a tool designed to measure work engagement across three dimensions of vigor (VI), dedication (DE), and absorption (AB). The 9-item version used in this study (e.g., “My job inspires me”), consisting of three items per dimension, is an adaptation to the Italian language and context [39] of the tool developed by Schaufeli, Bakker & Salanova [47]. Items assess the frequency of work-related experiences, ranging from 0 (“Never”) to 6 (“Always”). Consistently with the literature this study found excellent reliability values for both total engagement (ω = 0.95) and the various dimensions (ω VI = 0.92, ω DE = 0.87, ω AB = 0.88).

*Satisfaction with Job – General (SJG-5) –* The Satisfaction with Job-General-5 (or SJG-5) is a tool designed to measure job satisfaction using a seven-point Likert scale (1 = “Completely disagree”, 7 = “Completely agree”) developed by Hackman and Oldham [48] and adapted in Italian by Barbaranelli et al. [49] (e.g., “People who do this job often think about leaving it”). The instrument in this study showed high reliability (α and ω = 0.85).

*Satisfaction with Life (SWLS) –* The Satisfaction with Life Scale (SWLS) is a tool developed by Diener and colleagues [50] and validated in Italian for the general population by Di Fabio and Gori [51]. The tool, assessing cognitive judgments of life satisfaction, consists of 5 items (e.g., “In many ways, my life is close to my ideal”) on which the participant expresses their degree of agreement using a 7-point Likert scale (1 = “Strongly disagree”, 4 = “Neither agree nor disagree”, 7 = “Strongly agree”). The instrument in this study showed excellent reliability (ω = 0.92).

*Burnout Assessment Tool (BAT-12) –* The Burnout Assessment Tool (BAT-12), is a short version of the 23-item questionnaire developed by Schaufeli, De Witte and Desart [52], created by Hadžibajramović et al. [53] and adapted to Italian by Mazzetti et al. [54] (e.g., “At work, I feel mentally exhausted”). The instrument measures the frequency of burnout symptoms related to the dimensions of exhaustion (EX), mental distance (MD), cognitive impairment (CI), and emotional impairment (EI) using a five-point scale ranging from 1 (“Never”) to 5 (“Always”). In this study the instrument showed excellent levels of reliability for both the Burnout factor (ω = 0.91) and the individual dimensions (ω EX = 0.86; ω MD = 0.86; ω CI = 0.85; ω EI = 0.85).

*Sociodemographic questionnaire –* The final part of the questionnaire collected sociodemographic information, including gender, age group, sector of employment, contractual status, level of education, average monthly net income from employment, geographical location, size of the organization, position held, and length of service.

### Data analysis

Data were analyzed through R version 4.5.1. Confirmatory Factor Analysis path diagrams were produced with the support of IBM SPSS Amos V.30. Data screening procedures resulted in the removal of eight participants who failed attention checks and additional participants identified as multivariate outliers using Mahalanobis distance (p < .001) [55], yielding a final sample of 673 individuals. Item-level normality was assessed through skewness and kurtosis values within the acceptable ranges of ±2 [56].

Means and standard deviations were calculated to provide a general description of the sample’s responses and to summarize the overall distribution of the study variables. Reliability was assessed using Cronbach’s Alpha and McDonald’s Omega for each relevant dimension in the study. While a reliability of 0.70 is generally considered acceptable and 0.80 good in literature, it is important to interpret these values in the context of the specific purpose and characteristics of the given measure [57]. To examine the factor structure of the 29-item version of the PFW scale, confirmatory factor analyses were conducted using maximum likelihood estimation to test four theoretical models: single-factor, correlated nine-factor, hierarchical, and bifactor structures. Similarly, the 9-item version of the PF-W scale was assessed through single factor model. Model fit was evaluated using established criteria: comparative fit index (CFI) and Tucker–Lewis index (TLI) ≥.95 (excellent) and ≥.90 (acceptable), root mean square error of approximation (RMSEA) ≤.05 (excellent), ≤ .08 (acceptable) and ≤.10 (mediocre), and standardized root mean square residual (SRMR) ≤.08 [58,59]. For the bifactor model, percentage of uncontaminated correlations (PUC), explained common variance (ECV), and omega hierarchical (ωh) were examined to assess the appropriateness of total and subscale scoring.

To assess whether the measurement model functioned equivalently across groups, multi-group confirmatory factor analysis (MGCFA) was conducted for both the PFW-29 correlated model and the PFW-9 single factor model on education, age, gender, region, income, seniority, company size and work modality. Recommended thresholds for evaluating measurement invariance are well established in the literature. Following Chen [60], ΔCFI values of ≤ 0.010 and ΔRMSEA values of ≤ 0.015 are considered acceptable across all levels of invariance. For ΔSRMR, a value of ≤ 0.030 is acceptable when testing metric invariance (factor loadings), whereas stricter criteria of ≤ 0.010 apply for scalar invariance (intercepts) and residual invariance.

To ensure adequate group sizes for multigroup CFA, several sociodemographic variables were aggregated, and some very small categories were excluded from the analysis. Education was grouped into “no university degree” and “university degree”. Gender included only males and females, excluding non-binary and “prefer not to answer” responses. Region was grouped into North, Center and South/Islands, excluding respondents living abroad. Income was categorized as less than €1,500 versus €1,500 or more, excluding respondents who preferred not to answer. Company size was collapsed into three categories: micro-organizations, small-to-medium organizations, and large or public organizations. Finally, work modality was grouped as on-site versus hybrid/remote. Aggregation was guided by both theoretical and practical considerations, balancing interpretability with the need to maintain sufficient group sizes, ideally superior to 200 [61], for reliable invariance testing. Invariance conclusions should therefore be interpreted with reference to the specific categories as defined and may not generalize to more granular subgroups.

In addition to the confirmatory factor analysis, analysis within the item response theory framework was conducted to further examine item functioning of the PFW-9 at the item level. A graded response model (GRM) was used to assess difficulty and discrimination for each item, each representing a theoretical dimension of well-being. Item information functions and test information function plots were analyzed to evaluate the relative informativity of each item and of the questionnaire as a whole. Item discrimination parameters (*a*) were interpreted following Baker’s guidelines [62], where values below 0.65 indicate low discrimination, 0.65–1.34 moderate, and > 1.35 high discrimination. Threshold parameters (*b*) were expected to be ordered and approximately distributed between −3 and +3 along the latent continuum, as disordered thresholds suggest category functioning issues [62].

Item-level fit was assessed using the S-χ² statistic and its associated RMSEA(S-χ²). Given that the χ² test is highly sensitive to large sample sizes, a significance threshold of p < 0.01 was adopted. RMSEA(S-χ²) was used as a more stable indicator of fit. In IRT applications, RMSEA(S-χ²) values below .06 indicate good fit [63]. Local dependence (LD) among items was evaluated using Yen’s Q3 statistic [64], computed as the correlation between residuals from the unidimensional item response model. Following Christensen, Makransky & Horton [65], residual correlations were interpreted relative to the mean residual correlation, with Q3 values exceeding approximately 0.2 above the mean considered indicative of potential local dependence.

Convergent and discriminant validity were assessed through Pearson correlations between PERMA+4 dimensions and between PERMA+4 and established measures of work engagement (UWES-9), job satisfaction (SJG-5), life satisfaction (SWLS) and burnout (BAT-12). Effect sizes were interpreted using Cohen’s guidelines [66], with convergent validity supported by at least moderate correlations (r = .30) and discriminant validity supported by correlations below .85 [67]. Criterion validity was examined through correlations with work performance dimensions measured by the Individual Work Performance Questionnaire.

Incremental validity was tested using hierarchical multiple regression analyses. PERMA+4’s incremental contribution beyond the original PERMA dimensions was assessed by entering PERMA dimensions as assessed in the PFW-29 in the first block and the four additional dimensions in the second block. All regression assumptions were verified, including absence of multicollinearity (VIF < 10, r < .85), independence of errors (Durbin-Watson statistic between 1.5–2.5), normality of residuals, and homoscedasticity through visual inspection of diagnostic plots. Practical effect size was evaluated through Cohen’s *f²* statistic [66].

## Results

The results of the descriptive statistics and the reliability indices for the relevant instruments are shown in Table 4. PFW-29 showed a mean of 4.62 (SD = 0.87) in a 1–7 Likert scale, while PFW-9 displayed a mean of 6.78 (SD = 1.86) in a 0–10 Likert scale. Across the PFW-29 dimensions, mean scores ranged from 3.73 (SD = 1.53) for economic security to 5.13 (SD = 1.17) for engagement. The dimensions of the PFW-29 instrument consistently showed high levels of reliability (α and ω > .80), except for the accomplishment dimension that still showed acceptable results (α, ω = .77) and the physical environment dimension, which showed somewhat lower internal consistency (α = .64 and ω = .65) that should be contextualized within its conceptual breadth. Both the overall of PFW-29 (α, ω = .93) and the PFW-9 (α = .91; ω = .92) instruments showed excellent reliability values. Similarly, most of the other measures in the study showed good levels of reliability (α, ω > 0.80).

**Table 4:** Reliability, means, standard deviation and correlations between PFW, well-being and work outcomes measures.

	M	*SD*	N	1.	2.	3.	4.	5.	6.	7.	8.	9.	10.	11.
α				0.93	0.84	0.90	0.91	0.77	0.86	0.81	0.64	0.82	0.93	0.91
ω				0.93	0.85	0.91	0.92	0.77	0.87	0.82	0.66	0.83	0.94	0.92
1. PE	4.38	1.39	673	--										
2. EN	5.13	1.17	673	.51^**^	--									
3. REL	4.81	1.17	673	.47^**^	.31^**^	--								
4. MEAN	4.57	1.45	673	.71^**^	.42^**^	.38^**^	--							
5. ACC	4.93	1.09	673	.62^**^	.46^**^	.39^**^	.63^**^	--						
6. PH	4.97	1.14	673	.36^**^	.23^**^	.37^**^	.32^**^	.39^**^	--					
7. MIND	4.56	1.27	673	.68^**^	.42^**^	.46^**^	.63^**^	.64^**^	.49^**^	--				
8. ENV	4.35	1.30	673	.47^**^	.23^**^	.32^**^	.36^**^	.35^**^	.32^**^	.47^**^	--			
9. ES	3.73	1.53	673	.29^**^	.11^**^	.34^**^	.22^**^	.31^**^	.34^**^	.33^**^	.27^**^	--		
10. PFW-29	4.62	0.87	673	.82^**^	.58^**^	.67^**^	.75^**^	.76^**^	.63^**^	.82^**^	.61^**^	.54^**^	--	
11. PFW-9	6.78	1.86	673	.78**	.45**	.53**	.67**	.70**	.50**	.76**	.53**	.47**	.87**	--
SWLS	20.08	6.85	673	.54^**^	.32^**^	.43^**^	.42^**^	.52^**^	.47^**^	.53^**^	.37^**^	.51^**^	.67^**^	.64**
BAT	2.26	0.75	673	−.61^**^	−.34^**^	−.41^**^	−.49^**^	−.50^**^	−.43^**^	−.48^**^	−.33^**^	−.26^**^	−.62^**^	−.63**
SJG	22.02	6.28	673	.76^**^	.36^**^	.46^**^	.63^**^	.58^**^	.33^**^	.64^**^	.43^**^	.38^**^	.74^**^	.76**
IWPQ-TP	3.97	0.70	673	.40^**^	.26^**^	.31^**^	.36^**^	.60^**^	.34^**^	.35^**^	.24^**^	.23^**^	.50^**^	.52**
IWPQ-CP	3.57	0.72	673	.55^**^	.47^**^	.35^**^	.56^**^	.63^**^	.31^**^	.60^**^	.37^**^	.23^**^	.65^**^	.64**
IWPQ -CW	2.65	0.84	673	−.30^**^	−.08^*^	−.17^**^	−.18^**^	−.18^**^	−.22^**^	−.24^**^	−.20^**^	−.13^**^	−.28^**^	−.35**
UWES	4.48	1.28	673	.85^**^	.55^**^	.46^**^	.73^**^	.73^**^	.67^**^	.40^**^	.47^**^	.33^**^	.83^**^	.83**

Notes. * indicates p < .05. ** indicates p < .01. PE = Positive Emotions; EN = Engagement; REL = Relationships; MEAN = Meaning; ACC = Accomplishment; PH = Physical Health; MIND = Mindset; ENV = Environment; ES = Economic Security. PFW-29 = Positive Functioning At Work Scale – 29 item, PFW-9 = Positive Functioning At Work Scale – 9 item, SWLS = Satisfaction with Life Scale, BAT = Burnout Assessment Tool, SJG = Satisfaction with Job (General), IWPQ-TP = Individual Work Performance – Task Performance, IWPQ-CP = Individual Work Performance – Contextual Performance, IWPQ-CW = Individual Work Performance – Counterproductive Work Behaviors, UWES = Utrecht Work Engagement Scale. SWLS and SJG M(SD) refer to the total score. α = Cronbach’s alpha, ω = McDonald’s omega.

### Confirmatory factor analysis

Regarding the factor structure of the PFW-29, the four tested models yielded results that align with existing literature. Both the correlated and hierarchical models demonstrated a good fit to the data, while the single-factor model exhibited a poor fit. The bifactor model showed the best model fit, illustrating how items from different dimensions contributed differently to the general and specific factors. For instance, the third item pertaining to positive emotions exhibited a strong standardized loading on the general factor (β = .82) and a smaller loading on the specific positive emotions factor (β = .38). Conversely, the second item of economic security showed a moderate loading on the general factor (β = .25) and a strong loading on the specific economic security factor (β = .86).

Bifactor-specific indices were examined to assess the contribution of the general well-being factor. The explained common variance (ECV = .48) indicated that approximately 48% of the variance shared among items was accounted for by the general factor, suggesting that domain-specific group factors captured a meaningful portion of the common variance alongside it. The percentage of uncontaminated correlations (PUC = 0.92) was high, signifying that most item correlations were uncontaminated by multidimensionality. According to Rodriguez, Reise, and Haviland [68], when PUC is this high, the magnitude of the ECV becomes less critical because the general factor in a bifactor model will be highly similar to the trait estimated in a unidimensional model, suggesting minimal parameter bias. Omega hierarchical (ωh = 0.86) indicated that approximately 86% of the variance in the unit-weighted total score was attributable to the general factor, suggesting that the total score can be interpreted as largely reflecting a single underlying construct while preserving the interpretive value of the subscales. Overall, these results support the use of both a total well-being score and dimension-level scores for organizational assessment.

**Fig 1:**
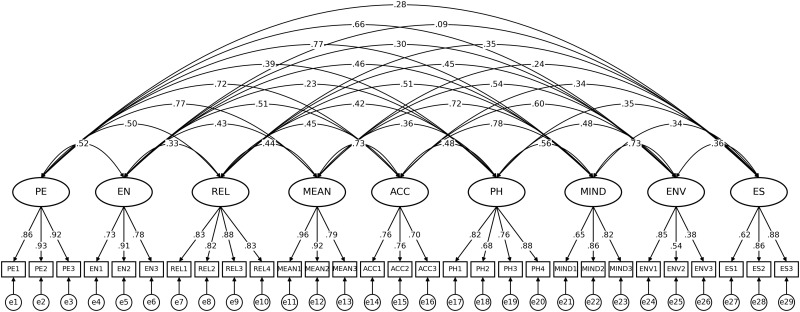
Correlated model.

**Fig 2:**
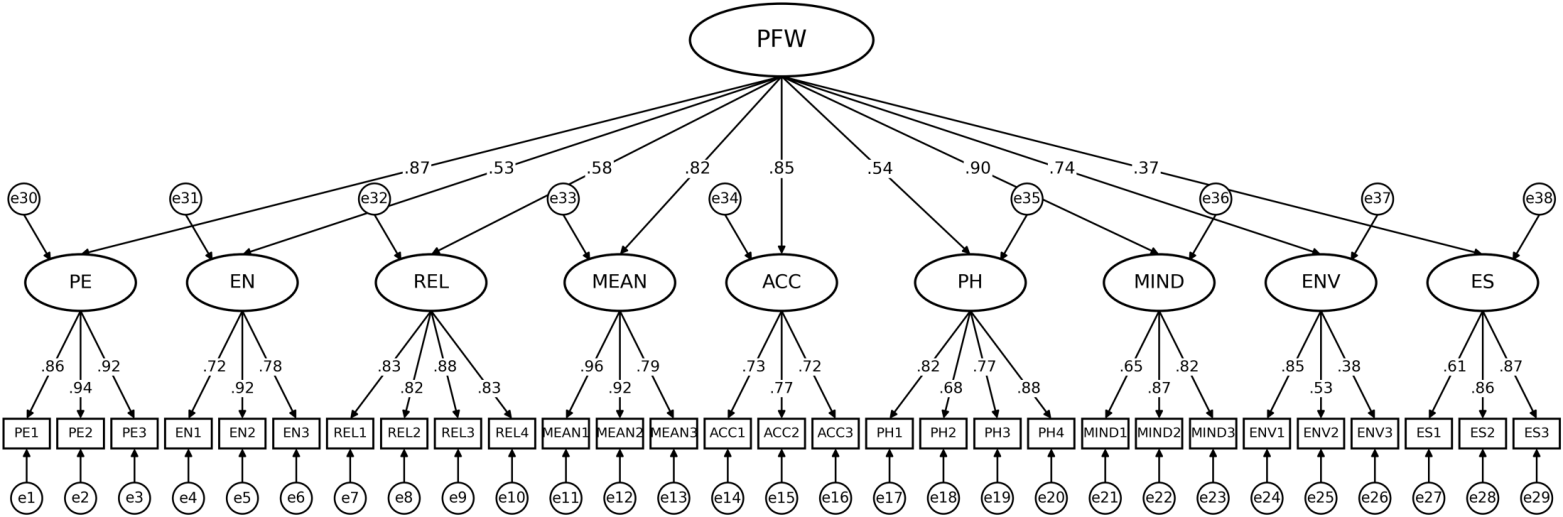
Hierarchical model.

**Fig 3:**
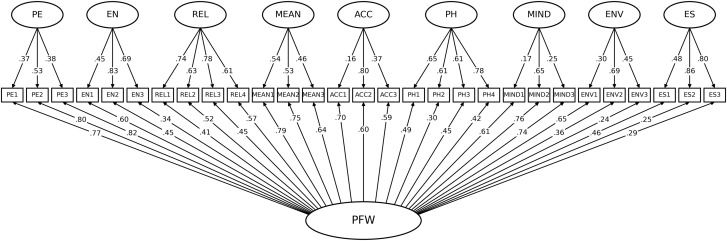
Bifactor model.

**Fig 4:**
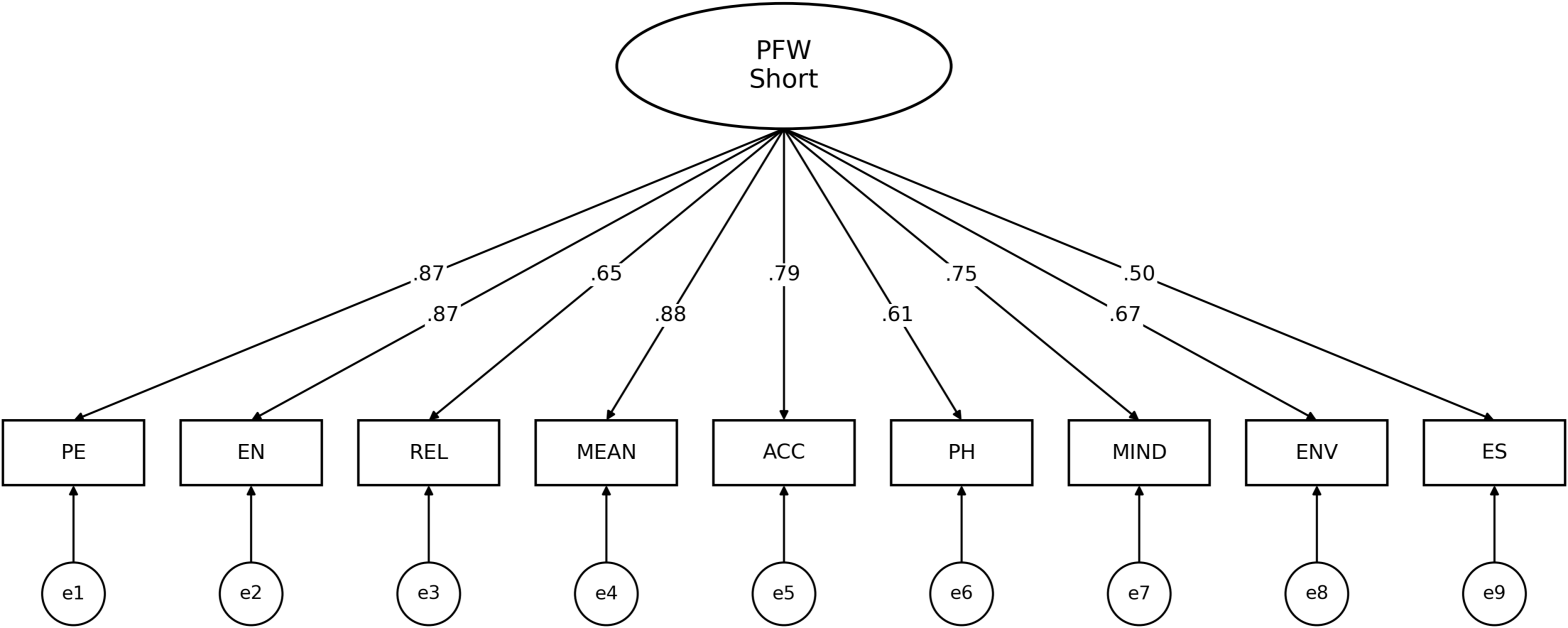
Single factor short version model.

A similar pattern is found in the PFW-9. Regarding the factorial structure, the model exhibited an acceptable fit to the data, although the RMSEA value was slightly elevated, suggesting the presence of latent complexity not fully captured by the model (χ²(27) = 197, p < .001; SRMR = .038; RMSEA = .097, 90% CI [.084,.110], p < .001; CFI = .954; TLI = .938). Factor loadings of the items on the main factor ranged from high (β > .70) to acceptable (β > .50). Specifically, positive emotions, engagement, and meaning exceeded .80, whereas economic security showed a lower loading, below .60. Figs 1–4 show the factor model diagrams for the good-fitting models of both PFW-29 and PFW-9 scales. Table 5 provides the summary of the model fit for the five models tested in our analysis.

**Table 5:** Fit indices of the model alternatives for the long and the short versions of the Positive Functioning At Work Scale.

Model	df	χ²	SRMR	RMSEA	90% CI RMSEA		CFI	TLI
					LB	UB		
One Factor	377	6064*	0.107	0.150	0.146	0.153	0.561	0.527
Hierarchical	368	1467*	0.076	0.067	0.063	0.070	0.915	0.906
Bifactor	350	1002*	0.051	0.053	0.049	0.056	0.950	0.942
Correlated	341	1287*	0.064	0.064	0.060	0.068	0.927	0.913
Short Single	27	197*	0.042	0.097	0.084	0.110	0.954	0.938

Notes. χ² = chi-square statistic; df = degrees of freedom; SRMR = Standardized Root Mean Square Residual; RMSEA = Root Mean Square Error of Approximation; CI = confidence interval; LB = lower bound; UB = upper bound; CFI = Comparative Fit Index; TLI = Tucker-Lewis Index. Short Single model refers to the short version of the scale; all other models refer to the long version of the scale. * indicates p < .001.

### Measurement invariance

Regarding measurement invariance, analyzed through MGCFA in relation to the correlated model of the PFW-29 instrument and the single factor model for PFW-9, configural, metric, scalar and strict variance were confirmed for most of the assessed grouped sociodemographic variables (i.e., age, gender, education, region, seniority, company size and work modality variables). An exception was observed for income in the PFW-9 scale, for which only metric invariance was supported. The failure to achieve invariance for the income variable in the PFW-9 may be attributable to the reduced number of items per dimension in the questionnaire, which increases sensitivity to group-specific residual differences that are averaged out in the full PFW-29 scale.

The measurement invariance results support the generalizability of the PFW scales’ functioning, as evidenced by the maintenance of acceptable fit indices across configural, metric, scalar and strict levels for most grouping variables. Thus, the scales measure the same constructs with equivalent factor loadings, item intercepts and residuals across almost all the aforementioned groups. S3–S18 Tables in supplementary materials provide the model fit statistics for the various MGCFA analyses.

### Item response theory analysis

The graded response model analysis conducted on the items of the PFW-9 scale (Table 6) indicated that almost all items demonstrated high discrimination ability (*a* > 1.34), with the sole exception of the item related to economic security, which still exhibited moderate discrimination (*a* = 1.16). Regarding the difficulty parameters (*b*), most items spanned a wide range of θ levels. However, in some cases, responses at the extremes were either too easy (*b*₁ > −2 and *b*₁₀ < 2) or too difficult (*b*₁ < −3 and *b*₁₀ > 3).

**Table 6:** Item parameters of the graded response model for the Positive Functioning at Work Scale – Short Version.

Item	a	b1	b2	b3	b4	b5	b6	b7	b8	b9	b10
Positive Emotions	3.67	−2.18	−1.92	−1.46	−1.07	−0.75	−0.49	0.02	0.64	1.39	2.11
Engagement	3.49	−2.41	−1.95	−1.45	−1.14	−0.79	−0.41	0.11	0.69	1.45	2.05
Relationships	1.79	−3.37	−2.84	−2.28	−1.81	−1.45	−0.78	−0.20	0.54	1.39	2.12
Meaning	3.56	−1.97	−1.58	−1.29	−0.92	−0.65	−0.31	0.14	0.58	1.16	1.79
Accomplishment	2.55	−2.50	−2.09	−1.70	−1.52	−1.08	−0.50	−0.01	0.52	1.26	1.94
Physical Health	1.52	−3.19	−2.60	−1.96	−1.44	−1.09	−0.54	0.04	0.76	1.53	2.48
Mindset	2.29	−1.53	−1.18	−0.78	−0.46	−0.16	0.27	0.73	1.22	1.78	2.38
Environment	1.75	−2.52	−2.01	−1.52	−1.11	−0.80	−0.33	0.18	0.74	1.46	2.31
Economic Security	1.16	−2.59	−1.94	−1.38	−0.84	−0.44	0.09	0.68	1.39	2.33	3.22

Notes: a = discrimination; b1-b10 = difficulty thresholds

Item-level fit highlighted excellent levels of RMSEA(S- χ²) with values ≤ 0.02 and non-significant S- χ² statistic. Local dependence [Q_3max_-Q_3mean_ = 0.208 -(-0.093) = 0.301] was found, but it pertained only to the mindset-economic security pair of items (r = 0.208). Fit results and the Q3 residuals matrix are presented in S19 and S20 Tables.

**Fig 5:**
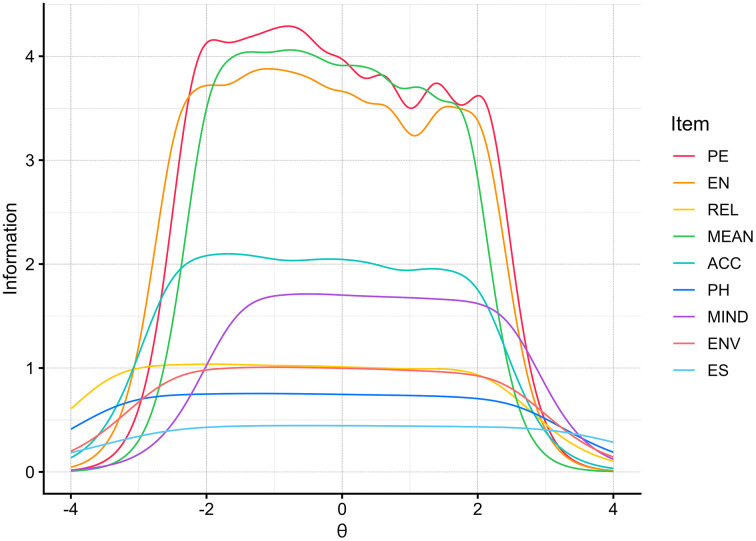
Item information function of the PFW-9.

**Fig 6:**
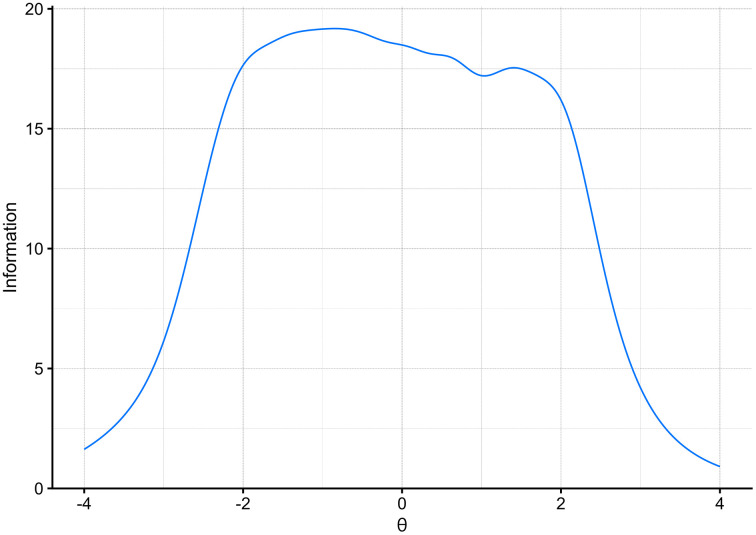
Test information function of the PFW-9.

These results were further corroborated by the Item Information Function (Fig 5), which indicated that the mindset dimension provided limited information for low-to-moderate θ levels. Physical health and economic security also contributed minimally compared to the other dimensions. Conversely, dimensions such as positive emotions, meaning, and engagement exhibited high informational value. Finally, the test information function (Fig 6) demonstrated that the instrument, in its entirety, delivers substantial information across a wide θ range.

### Convergent, discriminant, and criterion validity assessment

Correlation analyses were conducted to assess convergent, discriminant and criterion validity among relevant variables. Both latent and observed correlations between the PFW-29 dimensions highlighted significant and heterogeneous relationships between the questionnaire dimensions, supporting their discriminant validity (r < 0.85), while the absence of discriminant validity between PFW-29 and PFW-9 (r = 0.87) suggests that the two versions of the PFW scale measure the same construct.

Convergent and discriminant validity were supported between both the PFW-29 and PFW-9 and the UWES-9 (r = 0.83–0.83), SJG-5 (r = 0.74–0.76), SWLS-5 (r = 0.64–0.67), and BAT-12 (r = −0.62– − 0.63), with high correlations (r > 0.50) that remained below the redundancy threshold (r < 0.85). Furthermore, criterion validity was supported between both the PFW-29 and PFW-9 and the IWPQ-17 dimensions of task performance (r = 0.50–0.52), contextual performance (r = 0.64–0.65), and counterproductive work behaviors (r = −0.28– − 0.35), revealing strong relationships with task and contextual performance and a moderate relationship with counterproductive work behaviors. The correlation patterns are nearly identical between the two versions of the scale, supporting the similarity of their nomological networks. Table 4 provides further information about reliability and validity analysis.

### Incremental predictive validity assessment

Hierarchical multiple regressions were conducted, to analyze the incremental predictive validity of the PERMA+4 model compared to the original PERMA. With respect to task performance (see Table 7) the model including only the five original PERMA dimensions accounted for 37% of the variance, with accomplishment (β = 0.59, p < .001) and relationships (β = 0.08, p < .05) emerging as significant predictors. When the four additional dimensions were incorporated into the model, the explained variance rose to 39%. Although this increase was statistically significant, its practical significance was modest (ΔR² = 0.02; *f²* ≈ 0.03). In the final model, accomplishment (β = 0.60, p < .001), physical health (β = 0.14, p < .001), and mindset (β = −0.14, p < .01) remained significant, whereas the remaining dimensions did not contribute uniquely to the prediction of task performance.

**Table 7:** Incremental predictive validity of PERMA+4 for work performance beyond PERMA.

Task Performance						
Dimensions	PERMA			PERMA+4		
	**B**	**β**	**Sig.**	**B**	**β**	**Sig.**
Positive Emotions	.03	.06	.196	.04	.09	.086
Engagement	-.03	-.05	.21	-.02	-.04	.265
Relationships	.05	.08	.017*	.04	.07	.062
Meaning	-.03	-.06	.162	-.02	-.04	.362
Accomplishment	.38	.59	.000***	.38	.60	.000***
Physical Health				.09	.14	.000***
Mindset				-.08	-.14	.003**
Environment				.01	.01	.768
Economic Security				.00	.01	.797
**R2**	.37			.39		
**ΔR2**				.02*		
**Contextual Performance**						
**Dimensions**	**PERMA**			**PERMA+4**		
	**B**	**β**	**Sig.**	**B**	**β**	**Sig.**
Positive Emotions	.05	.09	.048*	-.00	-.00	.938
Engagement	.10	.17	.000***	.10	.16	.000***
Relationships	.03	.05	.146	.01	.01	.662
Meaning	.09	.18	.000***	.07	.14	.001**
Accomplishment	.24	.37	.000***	.20	.31	.000***
Physical Health				-.02	-.02	.438
Mindset				.13	.23	.000***
Environment				.04	.07	.031*
Economic Security				-.00	-.00	.948
**R2**	.47			.50		
**ΔR2**				.03**		
**Counterproductive Work Behaviors**						
**Dimensions**	**PERMA**			**PERMA+4**		
	**B**	**β**	**Sig.**	**B**	**β**	**Sig.**
Positive Emotions	-.20	-.34	.000***	-.20	-.32	.000***
Engagement	.08	.11	.009**	.08	.11	.011*
Relationships	-.03	-.05	.277	-.01	-.02	.707
Meaning	.05	.09	.089	.05	.09	.109
Accomplishment	-.08	-.10	.048*	-.06	-.08	.121
Physical Health				-.12	-.16	.000**
Mindset				.04	.06	.276
Environment				-.00	-.06	.194
Economic Security				.01	.02	.671
**R2**	.11			.13		
**ΔR2**				.02**		

Notes: B = unstandardized regression coefficient; β = standardized regression coefficient; R² = coefficient of determination; ΔR² = change in R². PERMA dimensions include Positive Emotions, Engagement, Relationships, Meaning, and Accomplishment. PERMA+4 includes all PERMA dimensions plus Physical Health, Mindset, Environment, and Economic Security. *p < .05. **p < .01. ***p < .001.

Regarding contextual performance (see Table 7), the model including only the five original PERMA dimensions accounted for 47% of the variance. Within this model, positive emotions (β = 0.09, p < .05), accomplishment (β = 0.37, p < .001), engagement (β = 0.17, p < .001), and meaning (β = 0.18, p < .001) emerged as significant predictors. When the four additional dimensions were introduced, the explained variance increased to 50%. Although this increment was statistically significant, its practical impact remained modest (ΔR² = 0.03, *f²* ≈ 0.06). In the final model, accomplishment (β = 0.31, p < .001), engagement (β = 0.16, p < .001), meaning (β = 0.14, p = .001), mindset (β = 0.23, p < .001), and environment (β = 0.07, p < .05) retained significance, whereas the remaining dimensions did not contribute uniquely to the prediction of contextual performance.

With respect to counterproductive work behaviors (see Table 7), the model including the five original PERMA dimensions explained 11% of the variance. Within this model, positive emotions (β = −0.34, p < .001), engagement (β = 0.11, p = < .01), and accomplishment (β = −0.10, p < .05) emerged as significant predictors. When the four additional PERMA+4 dimensions were incorporated, the explained variance increased to 13%. Although the increment was statistically significant, its practical effect was relatively small (ΔR² = 0.02, *f²* ≈ 0.02). In the final model, positive emotions (β = −0.32, p < .001), engagement (β = 0.11, p < .05), and physical health (β = −0.16, p < .000) remained significant, whereas the other dimensions did not show unique predictive value.

## Discussion

The validation of the PFW scales in Italy demonstrates robust psychometric properties that align with international findings, while also revealing culturally specific patterns. Italy, for instance, consistently exhibits the lowest average well-being scores across nearly all relevant dimensions compared to other international validations [9,15–19]. These findings are descriptive in nature and should be interpreted cautiously, as they have not been examined within a formal framework of cross-national measurement invariance. This pattern may nevertheless be considered in light of the Italian labor market context described above. The structural challenges characterizing the Italian work environment which ranges from economic insecurity and precarity to territorial disparities and widespread skills mismatch, may have collectively contributed to diminished well-being levels across multiple domains. Factor analysis of the PFW-29 confirmed the nine-dimensional structure of workplace well-being, with the bifactor model providing the best fit to the data and showing different levels of contribution of the dimensions’ items to the general and specific factors. For example, economic security tended to exhibit low loadings on the general factor and high loadings to the specific factor, while positive emotions showed the opposite pattern.

Similarly, the results of the PFW-9 single-factor model, that presents an excellent CFI of.954 but a slightly elevated RMSEA of.097 support the absence of strict unidimensionality and that, while the structure can be interpreted as reflecting a general factor of positive functioning at work, the dimensions exhibit a degree of relative independence and vary in their contribution to the construct. In particular, the IRT analysis confirmed that the PFW-9 provides substantial measurement information across the full latent trait range, although the levels of information varied across dimensions: positive emotions, meaning, and engagement items showed strong information, while economic security exhibited comparatively lower levels.

This pattern replicates the cross-cultural findings from the aforementioned cross-cultural validations [9,15–19], highlighting the same differential pattern of contribution of the dimensions to the general factor found in the previous studies. Dimensions such as positive emotions, meaning, and mindset appear to constitute the psychological core of the construct, being highly interrelated and operating at the individual level. Economic security, by contrast, may operate at a fundamentally different level, reflecting macro-structural conditions such as welfare policies, inflation, and labour market dynamics that lie beyond both individual and organizational control. Overall, these findings add further support to the theoretical argument, already emerging from previous cross-cultural validations, that workplace well-being operates both as a general construct and through distinct, measurable dimensions that can be targeted for intervention. Reliability analyses revealed that most dimensions achieved high standards consistent with previous cross-cultural validations, except for environment (ω = .65), whose lower reliability has also been consistently observed across multiple cultural contexts [9,15,18]. This pattern may derive from both the nature of the measured construct and the length of the scale. In particular, the measured dimension captures objective physical characteristics that can co-occur in any combination independently of one another, without necessarily converging on a single underlying latent construct. For example, the third item of the dimension (i.e., the one assessing proximity to green spaces) may reflect the geographical location of the workplace independently of the other environmental characteristics present. This may introduce variance unshared with the other two items, as suggested by the low factor loading (λ = 0.38) in the correlated model. This formative-like nature structurally limits internal consistency. Moreover, with only three heterogeneous items, the dimension is inherently constrained in its capacity to yield high reliability. The recurrence of this sub-optimal pattern across multiple cultural validations suggests it reflects inherent structural properties of the construct and the scale rather than sample-specific contingencies. Interestingly, environment shows markedly different reliability between the Italian (ω = .65) and Spanish (ω = .83) samples [16], despite these being arguably the most culturally similar contexts among the available validations.

Convergent validity was strongly supported through correlations with established well-being related measures such as work engagement, job and life satisfaction and burnout both in the brief and extended version of the scale, a pattern generally reported in the previous cross-cultural literature [9,15–19]. Furthermore, discriminant validity is supported for the different PFW dimensions, a pattern already present but not explicitly discussed in previous studies. Moreover, this study highlights a strong similarity in the nomological pattern and a high convergence (r = .87) between the two versions of the PFW scale, providing strong evidence that the brief and extended forms capture the same construct with comparable fidelity, supporting the use of the 9-item version as a reliable alternative when assessment time is limited.

The Engagement dimension may need further analysis and refinement, showing a weaker than expected relationship with the UWES, which has stronger relationships with other dimensions (e.g., positive emotions). The PFW engagement, that reflects a flow-like and narrower conception of engagement, may not reflect entirely the work engagement dimensions.

When compared directly to the original PERMA dimensions, the expanded framework provided small yet significant incremental explained variance for task performance, contextual performance and counterproductive work behaviors, highlighting the role of the further four dimensions present in the PERMA+4 model. These findings support Donaldson and Donaldson’s [9] theoretical argument that a more comprehensive framework better captures the complexity of workplace well-being.

The measurement invariance analysis, conducted on both the brief and extended versions of the scale, extends previous evidence limited to the two aforementioned American studies and the Spanish validation [9,15,16], confirming the invariance across age, work-role and education, but highlighting differences with the [15] work, which didn’t found full-invariance for gender. The present findings suggest that the Italian PFW scales operate equivalently across an array of different sociodemographic groups, supporting fair and equitable application in organizational settings. This finding is particularly important because it supports the high level of generalization of the PFW instruments, ensuring that well-being assessments provide unbiased evaluation across different demographic groups within Italian organizational contexts.

### Theoretical implications

The Italian validation of PERMA+4 contributes to the growing cross-cultural evidence supporting the expanded nine-dimensional framework beyond the original PERMA model. The generally consistent psychometric properties across the available validations suggest that the nine interrelated dimensions are able to capture a broad and coherent picture of workplace well-being across different cultural contexts, while the cross-cultural variability in engagement and environment indicates that not all dimensions function equivalently across all cultural settings. Moreover, the bifactor model results offer theoretical insights into the structure of workplace well-being, suggesting that positive functioning operates both as a general construct and through specific, measurable dimensions. This hierarchical conceptualization aligns with contemporary understanding of well-being as multifaceted [69], allowing targeted assessment and interventions. Furthermore, measurement invariance in the results suggest that the PFW scales function equivalently across different sociodemographic groups in the Italian context, and the high convergence and similar external validity pattern between the long and short forms suggests that both versions capture the same construct, supporting the practical flexibility of the framework.

### Practical implications

For organizational practitioners, the validated Italian PFW scales provide a psychometrically supported instrument for assessing employee well-being beyond deficit- or pathology-oriented approaches. Their multidimensional structure allows for a differentiated description of well-being profiles across the nine PERMA+4 dimensions, offering a more fine-grained alternative to global well-being indicators. The measurement invariance observed across the groups examined supports equitable score comparisons across subgroups, which is relevant for organizational contexts where profiles are compared across teams or demographic categories. While direct evidence for applied uses is lacking, the framework’s structure suggests several potential directions. The scales may support descriptive monitoring of workplace well-being and identification of areas of relative strength and weakness within employee groups. PERMA+4 profiles could help orient organizational attention toward specific dimensions (e.g., lower Environment scores may point toward workspace conditions). The framework’s strengths-based orientation may also complement deficit-focused approaches in coaching contexts, and the scales could serve as a self-report diagnostic to support employee awareness of their own well-being profile. Whether any of these applications improve well-being or performance remains to be tested through experimental or longitudinal designs.

### Limitations

Several limitations should be considered when interpreting the findings of this study. First, the cross-sectional design precludes causal inferences about the relationships between PERMA+4 dimensions and work outcomes. While the theoretical framework suggests that positive functioning contributes to improved performance, longitudinal evidence needed to establish temporal precedence and test causal hypotheses is still limited. For instance, a study conducted by Donaldson, Suchta & Donaldson [13] found positive functioning at work to be positively predictive of performance and negatively predictive of turnover intentions over time. However, to this date it remains the only longitudinal study examining these relationships. This gap underscores the need for longitudinal replications across different occupational and cultural contexts, including the Italian one, before firm causal conclusions can be drawn. The sample, while geographically diverse across Italian regions, was recruited through the Prolific platform, which may introduce selection bias toward individuals with higher digital literacy and engagement with online platforms. This limitation may affect generalizability to workers in traditional industries or those with limited technology access. Additionally, the sample was highly educated (69.1% holding at least a bachelor’s degree), which may not reflect the broader Italian workforce and could influence the factor structure and relationships observed.

The self-report methodology, while appropriate for measuring subjective well-being constructs, introduces potential common method bias that may inflate correlations between measures. Future research would benefit from incorporating multiple data sources, including supervisor ratings of performance and objective organizational metrics, to provide convergent evidence for the relationships observed.

The environment dimension showed the lowest reliability among all dimensions (ω = .65), a limitation consistent across multiple cultural validations [9,15,18]. The objective and formative-like nature of the construct, combined with a limited three-item pool covering heterogeneous aspects, structurally constrains internal consistency. Future research should address this through item expansion and subdivision into more homogeneous facets.

The study supported measurement invariance for both the PFW-9 and PFW-29 scales across several variables. However, the aggregation of demographic categories, while necessary to ensure adequate group sizes, has interpretive implications: broader groupings such as the binary income split or the macro-regional classification may mask subgroup differences that more refined categories would reveal. Moreover, the relatively small sample size per group (occasionally < 200 [61]) and the limited number of groups may have reduced statistical power to detect subtle violations of invariance, limiting the generalizability of the findings. However, the results are mostly coherent with past validations [9,16]. Moreover, this issue does not apply to the contrasting results found with the Cabrera [15] study, as the two considered gender categories were broad and comparable in size. In general, replicating these results in larger and more diverse samples would allow for more granular insights and sensitive testing.

The study did not examine test-retest reliability, limiting our understanding of the temporal stability of PERMA+4 scores. Given that well-being can fluctuate over time, establishing the scale’s stability properties is important for longitudinal applications and intervention evaluation.

Finally, the incremental validity analyses, while statistically significant, showed relatively modest effect sizes for some outcomes. Notably, this incremental contribution was largely driven by mindset and physical health, while economic security and environment showed limited unique predictive value. Given the already substantial variance explained by the original PERMA dimensions, these findings suggest that the practical added value of the extended framework is real but concentrated in specific dimensions, rather than uniformly distributed across all four additions. Practitioners should therefore weigh the added complexity of the full PERMA+4 assessment against the marginal predictive gains when selecting well-being measurement tools for organizational decision-making.

## Conclusions

This study validated both the brief and extended version of the PERMA+4 Positive Functioning at Work Scale in the Italian context, supporting the generalizability of the questionnaires among different work and sociodemographic settings. The study shows generally robust psychometric properties and strong relationships with important work outcomes. The findings contribute to the growing international evidence supporting the cross-cultural applicability of the PERMA+4 framework while revealing culturally specific patterns that enhance our understanding of workplace well-being. The Italian validation supports that positive functioning at work can be comprehensively assessed using the nine dimensions of PERMA+4, providing organizations with a theoretically grounded, empirically supported tool for employee well-being assessment.

The present findings, together with previous cross-cultural validations, provide cumulative evidence for the psychometric adequacy and practical utility of the PERMA+4 framework across different cultural contexts. For the Italian organizational psychology community, this validation provides confidence in implementing comprehensive well-being assessments that align with contemporary positive psychology principles.

Future research should address the study’s limitations through longitudinal designs, multi-source data collection, and further investigation of demographic moderators. Additionally, intervention research using PERMA+4 as both a diagnostic tool and outcome measure would provide valuable evidence for the framework’s practical utility in organizational settings.

## Supporting information

S1 Table Italian translation of the PFW-29. (DOCX)

S2 Table Italian translation of the PFW-9. (DOCX)

S3 Table Measurement invariance for education level: PFW-29. (DOCX)

S4 Table Measurement invariance for education level: PFW-9. (DOCX)

S5 Table Measurement invariance for age: PFW-29. (DOCX)

S6 Table Measurement invariance for age: PFW-9. (DOCX)

S7 Table Measure invariance for region: PFW-29. (DOCX)

S8 Table Measure invariance for region: PFW-9. (DOCX)

S9 Table Measure invariance for salary: PFW-29. (DOCX)

S10 Table Measure invariance for salary: PFW-9. (DOCX)

S11 Table Measure invariance for organization size: PFW-29. (DOCX)

S12 Table Measure invariance for organization size: PFW-9. (DOCX)

S13 Table Measure invariance for seniority: PFW-29. (DOCX)

S14 Table Measure invariance for seniority: PFW-9. (DOCX)

S15 Table Measure invariance for work modality: PFW-29. (DOCX)

S16 Table Measure invariance for work modality: PFW-9. (DOCX)

S17 Table Measure invariance for gender: PFW-29. (DOCX)

S18 Table Measure invariance for gender: PFW-9. (DOCX)

S19 Table Model fit for the PFW-9 graded response model. (DOCX)

S20 Table Q3 residual correlation matrix for the PFW-9 graded response model. (DOCX)

## References

[1] Seligman ME, Csikszentmihalyi M. Positive psychology. An introduction. Am Psychol. 2000;55(1):5–14. doi: 10.1037//0003-066x.55.1.5 11392865

[2] Diener E, Wirtz D, Tov W, Kim-Prieto C, Choi DW, Oishi S, et al. New well-being measures: Short scales to assess flourishing and positive and negative feelings. Soc Indic Res. 2010;97(2):143–56. doi: 10.1007/s11205-009-9493-y

[3] Keyes CLM. The mental health continuum: from languishing to flourishing in life. J Health Soc Behav. 2002;43(2):207–22. doi: 10.2307/3090197 12096700

[4] VanderWeele TJ, Trudel-Fitzgerald C, Allin P, Farrelly C, Fletcher G, Frederick DE, et al. Current recommendations on the selection of measures for well-being. Prev Med. 2020;133:106004. doi: 10.1016/j.ypmed.2020.106004 32006530

[5] Van Zyl LE, Gaffaney J, Van Der Vaart L, Dik BJ, Donaldson SI. The critiques and criticisms of positive psychology: a systematic review. J Posit Psychol. 2024;19(2):206–35. doi: 10.1080/17439760.2023.2178956

[6] Hendriks T, Warren MA, Schotanus-Dijkstra M, Hassankhan A, Graafsma T, Bohlmeijer E. How WEIRD are positive psychology interventions? A bibliometric analysis of randomized controlled trials on the science of well-being. J Posit Psychol. 2019;14(4):489–501. doi: 10.1080/17439760.2018.1484941

[7] Seligman MEP. Flourish: A Visionary New Understanding of Happiness and Well Being. New York, NY: Free Press. 2011.

[8] Kern ML. The Workplace PERMA Profiler [Internet]. 2014 [cited 2025 Jul 26]. Available from: http://www.peggykern.org/uploads/5/6/6/7/56678211/workplace_perma_profiler_102014.pdf

[9] Donaldson SI, Donaldson SI. The Positive Functioning at Work Scale: Psychometric Assessment, Validation, and Measurement Invariance. J well-being assess. 2020;4(2):181–215. doi: 10.1007/s41543-020-00033-1

[10] Donaldson SI, Dubin M. Flow 2.0: Optimal Experience in a Complex World: Honoring Mihaly Csikszentmihalyi’s Legacy. Hoboken, NJ: Wiley. 2025.

[11] Donaldson SI, Donaldson SI, McQuaid M, Kern ML. The PERMA 4 short scale: A cross-cultural empirical validation using item response theory. Int J Appl Posit Psychol. 2023;8(3):555–69. doi: 10.1007/s41042-023-00110-9

[12] Donaldson SI, Donaldson SI, McQuaid M, Kern ML. Systems-informed PERMA 4: Measuring well-being and performance at the employee, team, and supervisor levels. Int J Appl Posit Psychol. 2024;9(2):1153–66. doi: 10.1007/s41042-024-00177-y

[13] Donaldson SI, Suchta M, Donaldson SI. PERMA 4 well-being predicts the future: longitudinal evidence for employee positive functioning and performance. J Posit Psychol. 2025:1–6. doi: 10.1080/17439760.2025.2542236

[14] Cabrera V, Donaldson SI. PERMA to PERMA 4 building blocks of well-being: A systematic review of the empirical literature. J Posit Psychol. 2024;19(3):510–29. doi: 10.1080/17439760.2023.2208099

[15] Cabrera V. PERMA 4 building blocks of well-being: A mixed-methods exploration of mechanisms & conditions that enable the subjective well-being of workers. Claremont, CA: Claremont Graduate University. 2024.

[16] García-Selva A, Neipp M-C, Solanes-Puchol Á, Martín-Del-Río B, Donaldson SI. The PERMA+4 Positive Functioning at Work Scale: Spanish Adaptation and Validation. Psicothema. 2024;36(3):257–66. doi: 10.7334/psicothema2023.341 39054820

[17] Lorenz T, Ho J, Beyer M, Hagitte L. Measuring PERMA+4: validation of the German version of the Positive Functioning at Work Scale. Front Psychol. 2023;14:1231299. doi: 10.3389/fpsyg.2023.1231299 37637923 PMC10448252

[18] Demirci İ, Keskin Ö. Assessing employees’ positive functioning at work: adaptation and validation of the PERMA 4 scales into Turkish. Int J Appl Posit Psychol. 2025;10(3):54. doi: 10.1007/s41042-025-00248-8

[19] Smolyanov A, Gurieva S. The Russian version of the «PERMA 4 Short Scale». Psychol J High Sch Econ. 2025;22(2):261–87. doi: 10.17323/1813-8918-2025-2-261-287

[20] Organisation for Economic Co-operation and Development. OECD Skills Strategy Diagnostic Report: Italy 2017. Paris: OECD Publishing. 2018.

[21] Mandrone E, Pastore F, Quintano C, Radicchia D, Rocca A. Determinants and wage effects of overeducation in Italy. A comparison of five indicators of educational mismatch. Sinappsi. 2022;XII(3):130–55. doi: 10.53223/Sinappsi_2022-03-7

[22] Koopmans L, van den Tooren M, Preenen P. Long-term effects of on-the-job skills (mis)match on employee wellbeing and employability: a 7-wave longitudinal study. Front Psychol. 2025;16:1591769. doi: 10.3389/fpsyg.2025.1591769 41479979 PMC12753319

[23] Cuccu L, Royuela V, Scicchitano S. Navigating the precarious path: Understanding the dualisation of the Italian labour market through the lens of involuntary part-time employment. Papers in Regional Science. 2024;103(6):100061. doi: 10.1016/j.pirs.2024.100061

[24] Salvati L, Tridico P. Real wages and productivity: a lesson from Italy, 1980–2023. Struct Change Econ Dyn. 2025;75:261–72. doi: 10.1016/j.strueco.2025.08.004

[25] Moscone F, Tosetti E, Vittadini G. The impact of precarious employment on mental health: The case of Italy. Soc Sci Med. 2016;158:86–95. doi: 10.1016/j.socscimed.2016.03.008 27115334

[26] Panichella N. Geographical Origin, Internal Migration and Labour Market Attainment: An Analysis of Employment Opportunities and Class Attainment Among Italian Men and Women. Population Space and Place. 2024;31(1). doi: 10.1002/psp.2874

[27] D’Adamo I, Di Carlo C, Gastaldi M, Uricchio AF. Equitable and sustainable well-being indicators: A study of Italian regional disparities towards sustainable development. Sustain Dev. 2024;32(5):5538–49. doi: 10.1002/sd.2985

[28] Menon C, Vermeulen W. What drives regional productivity differentials? Evidence from firm-level data in Italy and Spain. OECD Local Economic and Employment Development (LEED) Papers, No. 2026/03. Paris: OECD Publishing; 2026. 10.1787/8fba4b12-en

[29] Coppens E, Hogg B, Greiner BA, Paterson C, de Winter L, Mathieu S, et al. Promoting employee wellbeing and preventing non-clinical mental health problems in the workplace: a preparatory consultation survey. J Occup Med Toxicol. 2023;18(1):17. doi: 10.1186/s12995-023-00378-2 37582790 PMC10426174

[30] Aust B, Leduc C, Cresswell-Smith J, O’Brien C, Rugulies R, Leduc M, et al. The effects of different types of organisational workplace mental health interventions on mental health and wellbeing in healthcare workers: a systematic review. Int Arch Occup Environ Health. 2024;97(5):485–522. doi: 10.1007/s00420-024-02065-z 38695906 PMC11130054

[31] De Angelis M, Giusino D, Nielsen K, Aboagye E, Christensen M, Innstrand ST, et al. H-WORK Project: Multilevel Interventions to Promote Mental Health in SMEs and Public Workplaces. Int J Environ Res Public Health. 2020;17(21):8035. doi: 10.3390/ijerph17218035 33142745 PMC7662282

[32] Cameron KS, Dutton JE, Quinn RE. An introduction to positive organizational scholarship. Positive Organizational Scholarship: Foundations of a New Discipline. San Francisco, CA: Berrett-Koehler. 2003. p. 3–13.

[33] Di Fabio A. The Psychology of Sustainability and Sustainable Development for Well-Being in Organizations. Front Psychol. 2017;8:1534. doi: 10.3389/fpsyg.2017.01534 28974935 PMC5610694

[34] Di Fabio A, Kenny ME. Promoting Well-Being: The Contribution of Emotional Intelligence. Front Psychol. 2016;7:1182. doi: 10.3389/fpsyg.2016.01182 27582713 PMC4987330

[35] Di Fabio A. Positive Relational Management for Healthy Organizations: Psychometric Properties of a New Scale for Prevention for Workers. Front Psychol. 2016;7:1523. doi: 10.3389/fpsyg.2016.01523 27790163 PMC5061777

[36] Di Fabio A. Flourishing Scale at Work: Psychometric Properties. Counseling. 2022;15:107–15. doi: 10.14605/CS1512207

[37] Giuntoli L, Ceccarini F, Sica C, Caudek C. Validation of the Italian Versions of the Flourishing Scale and of the Scale of Positive and Negative Experience. Sage Open. 2017;7(1). doi: 10.1177/2158244016682293

[38] Angelini G, Buonomo I, Benevene P, Consiglio P, Romano L, Fiorilli C. The Burnout Assessment Tool (BAT): A Contribution to Italian Validation with Teachers’. Sustainability. 2021;13(16):9065. doi: 10.3390/su13169065

[39] Balducci C, Fraccaroli F, Schaufeli WB. Psychometric properties of the Italian version of the Utrecht Work Engagement Scale (UWES-9): A cross-cultural analysis. Eur J Psychol Assess. 2010;26(2). doi: 10.1027/1015-5759/a000020

[40] Wild D, Grove A, Martin M, Eremenco S, McElroy S, Verjee-Lorenz A, et al. Principles of Good Practice for the Translation and Cultural Adaptation Process for Patient-Reported Outcomes (PRO) Measures: report of the ISPOR Task Force for Translation and Cultural Adaptation. Value Health. 2005;8(2):94–104. doi: 10.1111/j.1524-4733.2005.04054.x 15804318

[41] Cruchinho P, López-Franco MD, Capelas ML, Almeida S, Bennett PM, Miranda da Silva M, et al. Translation, Cross-Cultural Adaptation, and Validation of Measurement Instruments: A Practical Guideline for Novice Researchers. J Multidiscip Healthc. 2024;17:2701–28. doi: 10.2147/JMDH.S419714 38840704 PMC11151507

[42] Douglas BD, Ewell PJ, Brauer M. Data quality in online human-subjects research: Comparisons between MTurk, Prolific, CloudResearch, Qualtrics, and SONA. PLoS ONE. 2023;18(3):e0279720. doi: 10.1371/journal.pone.0279720 PMC10013894 36917576

[43] Istituto Nazionale di Statistica (ISTAT). La struttura delle retribuzioni in Italia [Internet]. Roma: ISTAT; 2025 [cited 2025 Jul 26]. Available from: https://www.istat.it/wp-content/uploads/2025/01/REPORT_STRUTTURA_RETRIBUZIONI_2022.pdf

[44] Istituto Nazionale di Statistica (ISTAT). Popolazione residente per sesso, età e stato civile al 1° gennaio 2025 [Internet]. Roma: ISTAT; 2025 [cited 2025 Jul 26]. Available from: https://demo.istat.it/app/?i=POS&l=it

[45] Koopmans L, Bernaards CM, Hildebrandt VH, van Buuren S, van der Beek AJ, de Vet HCW. Improving the Individual Work Performance Questionnaire using Rasch analysis. J Appl Meas. 2014;15(2):160–75. 24950534

[46] Platania S, Morando M, Gruttadauria SV, Koopmans L. The Individual Work Performance Questionnaire: Psychometric Properties of the Italian Version. Eur J Investig Health Psychol Educ. 2023;14(1):49–63. doi: 10.3390/ejihpe14010004 38248124 PMC10814648

[47] Schaufeli WB, Bakker AB, Salanova M. The measurement of work engagement with a short questionnaire: a cross-national study. Educ Psychol Meas. 2006;66(4):701–16. doi: 10.1177/0013164405282471

[48] Hackman JR, Oldham GR. Development of the Job Diagnostic Survey. J Appl Psychol. 1975;60(2):159–70. doi: 10.1037/h0076546

[49] Barbaranelli C, Bortone I, Di Matteo F. Measuring job satisfaction: an empirical contribution. G Ital Psicol. 2010;37(1):159–80. doi: 10.1421/3217

[50] Diener E, Emmons RA, Larsen RJ, Griffin S. The Satisfaction With Life Scale. J Pers Assess. 1985;49(1):71–5. doi: 10.1207/s15327752jpa4901_13 16367493

[51] Di Fabio A, Gori A. Satisfaction with Life Scale Among Italian Workers: Reliability, Factor Structure and Validity through a Big Sample Study. Sustainability. 2020;12(14):5860. doi: 10.3390/su12145860

[52] Schaufeli WB, De Witte H, Desart S. Burnout Assessment Tool (BAT)—development, validity, and reliability. Int J Environ Res Public Health. 2020;17(24):9495. doi: 10.3390/ijerph17249495 33352940 PMC7766078

[53] Hadžibajramović E, Schaufeli W, De Witte H. Shortening of the Burnout Assessment Tool (BAT)-from 23 to 12 items using content and Rasch analysis. BMC Public Health. 2022;22(1):560. doi: 10.1186/s12889-022-12946-y 35313849 PMC8939057

[54] Mazzetti G, Consiglio C, Santarpia FP, Borgogni L, Guglielmi D, Schaufeli WB. Italian Validation of the 12-Item Version of the Burnout Assessment Tool (BAT-12). Int J Environ Res Public Health. 2022;19(14):8562. doi: 10.3390/ijerph19148562 35886414 PMC9322735

[55] Kline RB. Principles and Practice of Structural Equation Modeling. 4th ed. New York, NY: Guilford Press. 2016.

[56] George D, Mallery P. SPSS for Windows Step by Step: A Simple Guide and Reference, 17.0 Update. 10th ed. Boston, MA: Allyn & Bacon. 2010.

[57] Dunn TJ, Baguley T, Brunsden V. From alpha to omega: a practical solution to the pervasive problem of internal consistency estimation. Br J Psychol. 2014;105(3):399–412. doi: 10.1111/bjop.12046 24844115

[58] Brown TA. Confirmatory Factor Analysis for Applied Research. 2nd ed. New York, NY: Guilford Press. 2015.

[59] Hu L, Bentler PM. Cutoff criteria for fit indexes in covariance structure analysis: Conventional criteria versus new alternatives. Structural Equation Modeling: A Multidisciplinary Journal. 1999;6(1):1–55. doi: 10.1080/10705519909540118

[60] Chen FF. Sensitivity of Goodness of Fit Indexes to Lack of Measurement Invariance. Structural Equation Modeling: A Multidisciplinary Journal. 2007;14(3):464–504. doi: 10.1080/10705510701301834

[61] Cheung GW, Rensvold RB. Evaluating Goodness-of-Fit Indexes for Testing Measurement Invariance. Structural Equation Modeling: A Multidisciplinary Journal. 2002;9(2):233–55. doi: 10.1207/s15328007sem0902_5

[62] Baker FB. The Basics of Item Response Theory. 2nd ed. Portsmouth, NH: Heinemann. 2001.

[63] Zein RA, Akhtar H. Getting started with the graded response model: An introduction and tutorial in R. Int J Psychol. 2025;60(1):e13265. doi: 10.1002/ijop.13265 39530209 PMC11626089

[64] Yen WM. Effects of local item dependence on the fit and equating performance of the three-parameter logistic model. Appl Psychol Meas. 1984;8(2):125–45. doi: 10.1177/014662168400800201

[65] Christensen KB, Makransky G, Horton M. Critical Values for Yen’s Q3: Identification of Local Dependence in the Rasch Model Using Residual Correlations. Appl Psychol Meas. 2017;41(3):178–94. doi: 10.1177/0146621616677520 29881087 PMC5978551

[66] Cohen J. Set correlation and contingency tables. Appl Psychol Meas. 1988;12(4):425–34.

[67] Campbell DT, Fiske DW. Convergent and discriminant validation by the multitrait-multimethod matrix. Psychol Bull. 1959;56(2):81–105. doi: 10.1037/h0046016 13634291

[68] Rodriguez A, Reise SP, Haviland MG. Evaluating bifactor models: Calculating and interpreting statistical indices. Psychol Methods. 2016;21(2):137–50. doi: 10.1037/met0000045 26523435

[69] Gallagher MW, Lopez SJ, Preacher KJ. The hierarchical structure of well-being. J Pers. 2009;77(4):1025–50. doi: 10.1111/j.1467-6494.2009.00573.x 19558444 PMC3865980

