# Supplementary material for: Evaluating employees’ positive functioning at work: Adaptation and validation of the PERMA+4 scales in the Italian context
Source: PLoS One. 2026 Aug 13;21(8):e0355712. doi: 10.1371/journal.pone.0355712 (PMC13472440; doi:10.1371/journal.pone.0355712)
Supplement: Supplementary file 1 [file pone.0355712.s001.docx]

| **Table S1**. Italian translation of the PFW-29 | | |
| --- | --- | --- |
| **Item** | **Italian Translation of the Items** | **English Original version of the items** |
| PE-1 | Provo gioia durante una tipica giornata di lavoro | I feel joy in a typical workday |
| PE-2 | Globalmente, sono entusiasta del mio lavoro | Overall, I feel enthusiastic about my work |
| PE-3 | Amo il mio lavoro | I love my job |
| EN-1 | Solitamente mi lascio assorbire mentre lavoro su qualcosa che sfida le mie capacità | I typically become absorbed while I am working on something that challenges my abilities |
| EN-2 | Perdo la cognizione del tempo mentre faccio qualcosa che mi piace a lavoro | I lose track of time while doing something I enjoy at work |
| EN-3 | Quando lavoro su qualcosa che mi piace, dimentico quello che ho intorno | When I am working on something I enjoy, I forget everything else around me |
| REL-1 | Posso contare sul supporto dei miei colleghi se ne ho bisogno | I can receive support from coworkers if I need it |
| REL-2 | Mi sento apprezzato/a dai miei colleghi/e | I feel appreciated by my coworkers |
| REL-3 | Mi fido dei miei colleghi/e | I trust my colleagues |
| REL-4 | I miei colleghi e/o le mie colleghe tirano fuori il meglio di me | My colleagues bring out my best self |
| MEAN-1 | Il mio lavoro è significativo | My work is meaningful |
| MEAN-2 | Capisco cosa rende il mio lavoro significativo | I understand what makes my job meaningful |
| MEAN-3 | Il lavoro che faccio ha un fine superiore | The work I do serves a greater purpose |
| ACC-1 | Fisso degli obiettivi che mi aiutano a raggiungere le mie aspirazioni lavorative | I set goals that help me achieve my career aspirations |
| ACC-2 | Solitamente raggiungo ciò che mi prefiggo nel mio lavoro | I typically accomplish what I set out to do in my job |
| ACC-3 | Sono perlopiù soddisfatto/a della mia performance a lavoro | I am generally satisfied with my performance at work |
| PH-1 | Di solito mi sento fisicamente in salute | I typically feel physically healthy |
| PH-2 | Mi ammalo raramente | I am rarely sick |
| PH-3 | Di solito posso superare le fonti di stress fisico (e.g., insonnia, infortuni, problemi di vista etc.) | I can typically overcome sources of physical distress (e.g., insomnia, injuries, vision issues, etc.) |
| PH-4 | Sento di avere il controllo della mia salute fisica | I feel in control of my physical health |
| MIND-1 | Credo di poter migliorare le mie capacità lavorative attraverso un forte impegno | I believe I can improve my job skills through hard work |
| MIND-2 | Credo che il mio lavoro mi permetterà di crescere in futuro | I believe my job will allow me to develop in the future |
| MIND-3 | Ho un futuro brillante all'interno dell'organizzazione per cui lavoro | I have a bright future at my current work organization |
| ENV-1 | Lo spazio fisico in cui lavoro (i.e., ufficio) mi permette di concentrarmi | My physical work environment (e.g., office space) allows me to focus on my work |
| ENV-2 | C'è molta luce naturale nel mio luogo di lavoro | There is plenty of natural light in my workplace |
| ENV-3 | Posso facilmente accedere a spazi verdi dal mio ambiente lavorativo (e.g., parchi, mare, montagne etc.) | I can conveniently access nature in my work environment (e.g., parks, oceans, mountains, etc.) |
| ES-1 | Mi sento a mio agio coi miei attuali guadagni | I am comfortable with my current income |
| ES-2 | Potrei perdere diversi mesi di paga a causa di una malattia importante, e avere comunque la mia sicurezza economica | I could lose several months of pay due to serious illness, and still have my economic security |
| ES-3 | Nel caso di un'emergenza finanziaria, ho risparmi sufficienti | In the event of a financial emergency, I have adequate savings |
| Notes: PE = Positive Emotions; EN = Engagement; REL = Relationships; MEAN = Meaning; ACC = Accomplishment; PH = Physical Health; MIND = Mindset; ENV = Environment; ES = Economic Security. For the PFW-29, responses were recorded on a 7-point Likert scale ranging from 1 (Strongly disagree) to 7 (Strongly agree). | | |
