# Supplementary material for: Evaluating employees’ positive functioning at work: Adaptation and validation of the PERMA+4 scales in the Italian context
Source: PLoS One. 2026 Aug 13;21(8):e0355712. doi: 10.1371/journal.pone.0355712 (PMC13472440; doi:10.1371/journal.pone.0355712)
Supplement: Supplementary file 2 [file pone.0355712.s002.docx]

| **Table S2***.* Italian translation of the PFW-9 | | |
| --- | --- | --- |
| **Item** | **Italian Translation of the Item** | **English Original version of the items** |
| PE | Mi sono sentito positivo a lavoro | I felt positive at work. |
| EN | Sono stato profondamente assorto e interessato al mio lavoro | I was deeply engaged and interested in my work. |
| REL | Sono stato incoraggiante e supportivo verso gli altri | I was encouraging and supportive of others. |
| MEAN | Ho sentito che il lavoro che facevo aveva valore e ne valeva la pena | I felt that the work I did was valuable and worthwhile. |
| ACC | Ho stabilito degli obiettivi chiari e li ho raggiunti | I set and achieved clear goals. |
| PH | Mi sono sentito fisicamente sano e forte | I felt physically healthy and strong. |
| MIND | Ho sentito di avere un futuro brillante all'interno dell'organizzazione per cui lavoro | I have a bright future at my current work organization |
| ENV | Lo spazio fisico in cui lavoro (i.e., ufficio) mi ha permesso di concentrarmi | My physical work environment (e.g., office space) allows me to focus on my work |
| ES | Mi sono sentito/a a mio agio coi miei attuali guadagni | I am comfortable with my current income |
| Notes: PE = Positive Emotions; EN = Engagement; REL = Relationships; MEAN = Meaning; ACC = Accomplishment; PH = Physical Health; MIND = Mindset; ENV = Environment; ES = Economic Security. For the PFW-9, responses were recorded on an 11-point Likert scale ranging from 0 (Strongly disagree) to 10 (Strongly agree). | | |
