# Supplementary material for: Evaluating employees’ positive functioning at work: Adaptation and validation of the PERMA+4 scales in the Italian context
Source: PLoS One. 2026 Aug 13;21(8):e0355712. doi: 10.1371/journal.pone.0355712 (PMC13472440; doi:10.1371/journal.pone.0355712)
Supplement: Supplementary file 3 [file pone.0355712.s003.docx]

| **Table S3.** Measurement invariance for education level: PFW-29 | | | | | | |
| --- | --- | --- | --- | --- | --- | --- |
| **Model** | **CFI** | **RMSEA** | **SRMR** | **ΔCFI** | **ΔRMSEA** | **ΔSRMR** |
| Configural | 0.9222 | 0.0664 | 0.0686 | *NA* | *NA* | *NA* |
| Metric | 0.9208 | 0.0661 | 0.0698 | 0.0014 | -0.0004 | 0.0012 |
| Scalar | 0.9178 | 0.0664 | 0.0704 | 0.0030 | 0.0003 | 0.0006 |
| Strict | 0.9188 | 0.0647 | 0.0701 | -0.0010 | -0.0017 | -0.0002 |
| Notes: Configural = same factor structure across groups; Metric = equality of factor loadings; Scalar = equality of item intercepts; Strict = equality of residual variances. CFI = Comparative Fit Index; RMSEA = Root Mean Square Error of Approximation; SRMR = Standardized Root Mean Square Residual. Δ values represent the absolute change in fit indices relative to the preceding invariance model. NA = not applicable. | | | | | | |
