# Supplementary material for: Evaluating employees’ positive functioning at work: Adaptation and validation of the PERMA+4 scales in the Italian context
Source: PLoS One. 2026 Aug 13;21(8):e0355712. doi: 10.1371/journal.pone.0355712 (PMC13472440; doi:10.1371/journal.pone.0355712)
Supplement: Supplementary file 9 [file pone.0355712.s009.docx]

| **Table S9.** Measure invariance for salary: PFW-29 | | | | | | |
| --- | --- | --- | --- | --- | --- | --- |
| **Model** | **CFI** | **RMSEA** | **SRMR** | **ΔCFI** | **ΔRMSEA** | **ΔSRMR** |
| Configural | 0.9236 | 0.0641 | 0.0687 | *NA* | *NA* | *NA* |
| Metric | 0.9215 | 0.0640 | 0.0711 | 0.0021 | -0.0001 | 0.0025 |
| Scalar | 0.9128 | 0.0666 | 0.0709 | 0.0087 | 0.0025 | -0.0003 |
| Strict | 0.9079 | 0.0671 | 0.0720 | 0.0049 | 0.0005 | 0.0011 |
| Notes: Configural = same factor structure across groups; Metric = equality of factor loadings; Scalar = equality of item intercepts; Strict = equality of residual variances. CFI = Comparative Fit Index; RMSEA = Root Mean Square Error of Approximation; SRMR = Standardized Root Mean Square Residual. Δ values represent the absolute change in fit indices relative to the preceding invariance model. NA = not applicable. | | | | | | |
