# Supplementary material for: Evaluating employees’ positive functioning at work: Adaptation and validation of the PERMA+4 scales in the Italian context
Source: PLoS One. 2026 Aug 13;21(8):e0355712. doi: 10.1371/journal.pone.0355712 (PMC13472440; doi:10.1371/journal.pone.0355712)
Supplement: Supplementary file 11 [file pone.0355712.s011.docx]

| **Table S11.** Measure invariance for organization size: PFW-29 | | | | | | |
| --- | --- | --- | --- | --- | --- | --- |
| **Model** | **CFI** | **RMSEA** | **SRMR** | **ΔCFI** | **ΔRMSEA** | **ΔSRMR** |
| Configural | 0.9130 | 0.0706 | 0.0725 | *NA* | *NA* | *NA* |
| Metric | 0.9115 | 0.0698 | 0.0748 | 0.0014 | -8,00E-04 | 0.0022 |
| Scalar | 0.9098 | 0.0692 | 0.0753 | 0.0017 | -6,00E-04 | 0.0006 |
| Strict | 0.9076 | 0.0683 | 0.0763 | 0.0022 | -9,00E-04 | 0.0010 |
| Notes: Configural = same factor structure across groups; Metric = equality of factor loadings; Scalar = equality of item intercepts; Strict = equality of residual variances. CFI = Comparative Fit Index; RMSEA = Root Mean Square Error of Approximation; SRMR = Standardized Root Mean Square Residual. Δ values represent the absolute change in fit indices relative to the preceding invariance model. NA = not applicable. | | | | | | |
