# Supplementary material for: Evaluating employees’ positive functioning at work: Adaptation and validation of the PERMA+4 scales in the Italian context
Source: PLoS One. 2026 Aug 13;21(8):e0355712. doi: 10.1371/journal.pone.0355712 (PMC13472440; doi:10.1371/journal.pone.0355712)
Supplement: Supplementary file 13 [file pone.0355712.s013.docx]

| **Table S13.** Measure invariance for seniority: PFW-29 | | | | | | |
| --- | --- | --- | --- | --- | --- | --- |
| **Model** | **CFI** | **RMSEA** | **SRMR** | **ΔCFI** | **ΔRMSEA** | **ΔSRMR** |
| Configural | 0.9173 | 0.0685 | 0.0721 | *NA* | *NA* | *NA* |
| Metric | 0.9150 | 0.0682 | 0.0737 | 0.0023 | -4,00E-04 | 0.0015 |
| Scalar | 0.9093 | 0.0691 | 0.0749 | 0.0057 | 1,00E-03 | 0.0013 |
| Strict | 0.9061 | 0.0686 | 0.0758 | 0.0032 | -6,00E-04 | 0.0008 |
| Notes: Configural = same factor structure across groups; Metric = equality of factor loadings; Scalar = equality of item intercepts; Strict = equality of residual variances. CFI = Comparative Fit Index; RMSEA = Root Mean Square Error of Approximation; SRMR = Standardized Root Mean Square Residual. Δ values represent the absolute change in fit indices relative to the preceding invariance model. NA = not applicable. | | | | | | |
