# Supplementary material for: Evaluating employees’ positive functioning at work: Adaptation and validation of the PERMA+4 scales in the Italian context
Source: PLoS One. 2026 Aug 13;21(8):e0355712. doi: 10.1371/journal.pone.0355712 (PMC13472440; doi:10.1371/journal.pone.0355712)
Supplement: Supplementary file 14 [file pone.0355712.s014.docx]

| **Table S14.** Measure invariance for seniority: PFW-9 | | | | | | |
| --- | --- | --- | --- | --- | --- | --- |
| **Model** | **CFI** | **RMSEA** | **SRMR** | **ΔCFI** | **ΔRMSEA** | **ΔSRMR** |
| Configural | 0.9483 | 0.1022 | 0.0433 | *NA* | *NA* | *NA* |
| Metric | 0.9462 | 0.0953 | 0.0624 | 0.0022 | -0.0069 | 0.0191 |
| Scalar | 0.9389 | 0.0941 | 0.0672 | 0.0073 | -0.0012 | 0.0048 |
| Strict | 0.9370 | 0.0887 | 0.0677 | 0.0018 | -0.0054 | 0.0005 |
| Notes: Configural = same factor structure across groups; Metric = equality of factor loadings; Scalar = equality of item intercepts; Strict = equality of residual variances. CFI = Comparative Fit Index; RMSEA = Root Mean Square Error of Approximation; SRMR = Standardized Root Mean Square Residual. Δ values represent the absolute change in fit indices relative to the preceding invariance model. NA = not applicable. | | | | | | |
