# Supplementary material for: Evaluating employees’ positive functioning at work: Adaptation and validation of the PERMA+4 scales in the Italian context
Source: PLoS One. 2026 Aug 13;21(8):e0355712. doi: 10.1371/journal.pone.0355712 (PMC13472440; doi:10.1371/journal.pone.0355712)
Supplement: Supplementary file 16 [file pone.0355712.s016.docx]

| **Table S16.** Measure invariance for work modality: PFW-9 | | | | | | |
| --- | --- | --- | --- | --- | --- | --- |
| **Model** | **CFI** | **RMSEA** | **SRMR** | **ΔCFI** | **ΔRMSEA** | **ΔSRMR** |
| Configural | 0.9451 | 0.1057 | 0.0429 | *NA* | *NA* | *NA* |
| Metric | 0.9458 | 0.0980 | 0.0472 | -0.0007 | -0.0077 | 0.0043 |
| Scalar | 0.9420 | 0.0954 | 0.0502 | 0.0038 | -0.0026 | 0.0030 |
| Strict | 0.9365 | 0.0939 | 0.0514 | 0.0055 | -0.0015 | 0.0012 |
| Notes: Configural = same factor structure across groups; Metric = equality of factor loadings; Scalar = equality of item intercepts; Strict = equality of residual variances. CFI = Comparative Fit Index; RMSEA = Root Mean Square Error of Approximation; SRMR = Standardized Root Mean Square Residual. Δ values represent the absolute change in fit indices relative to the preceding invariance model. NA = not applicable. | | | | | | |
