# Supplementary material for: Evaluating employees’ positive functioning at work: Adaptation and validation of the PERMA+4 scales in the Italian context
Source: PLoS One. 2026 Aug 13;21(8):e0355712. doi: 10.1371/journal.pone.0355712 (PMC13472440; doi:10.1371/journal.pone.0355712)
Supplement: Supplementary file 19 [file pone.0355712.s019.docx]

**Table S19.** Model fit for the PFW-9 graded response model

| **Item** | **S- χ²** | **df.S_- χ²** | **RMSEA.S-χ²** | **p.S- χ²** |
| --- | --- | --- | --- | --- |
| PE | 134.682 | 142 | 0.000 | 0.656 |
| EN | 183.674 | 146 | 0.020 | 0.019 |
| REL | 213.095 | 182 | 0.016 | 0.057 |
| MEAN | 190.157 | 160 | 0.017 | 0.052 |
| ACC | 157.208 | 158 | 0.000 | 0.503 |
| PH | 220.170 | 226 | 0.000 | 0.597 |
| MIND | 210.208 | 219 | 0.000 | 0.653 |
| ENV | 222.668 | 230 | 0.000 | 0.623 |
| ES | 275.305 | 274 | 0.003 | 0.466 |
| Notes: S-χ² = standardized chi-square statistic; df.S-χ² = degrees of freedom associated with the standardized chi-square; RMSEA.S-χ² = RMSEA based on the standardized chi-square statistic; p.S-χ² = p-value associated with the standardized chi-square test. PE = Positive Emotions; EN = Engagement; REL = Relationships; MEAN = Meaning; ACC = Accomplishment; PH = Physical Health; MIND = Mindset; ENV = Environment; ES = Economic Security. Values are reported for each PFW-9 item under the Graded Response Model. | | | | |
