# Supplementary material for: Evaluating employees’ positive functioning at work: Adaptation and validation of the PERMA+4 scales in the Italian context
Source: PLoS One. 2026 Aug 13;21(8):e0355712. doi: 10.1371/journal.pone.0355712 (PMC13472440; doi:10.1371/journal.pone.0355712)
Supplement: Supplementary file 20 [file pone.0355712.s020.docx]

**Table S20**. Q3 residual correlation matrix for the PFW-9 graded response model

| **Item** | **PE** | **EN** | **REL** | **MEAN** | **ACC** | **PH** | **MIND** | **ENV** | **ES** |
| --- | --- | --- | --- | --- | --- | --- | --- | --- | --- |
| PE | 1.000 |  |  |  |  |  |  |  |  |
| EN | -0.077 | 1.000 |  |  |  |  |  |  |  |
| REL | -0.081 | -0.066 | 1.000 |  |  |  |  |  |  |
| MEAN | -0.339 | -0.090 | -0.132 | 1.000 |  |  |  |  |  |
| ACC | -0.249 | -0.179 | -0.061 | -0.107 | 1.000 |  |  |  |  |
| PH | -0.106 | -0.234 | -0.080 | -0.140 | -0.028 | 1.000 |  |  |  |
| MIND | -0.148 | -0.299 | -0.155 | -0.093 | -0.094 | 0.045 | 1.000 |  |  |
| ENV | -0.151 | -0.235 | -0.105 | -0.204 | -0.019 | 0.103 | 0.094 | 1.000 |  |
| ES | -0.072 | -0.238 | 0.018 | -0.169 | -0.149 | 0.083 | **0.208** | 0.185 | 1.000 |
| Notes: Q3 residual correlation matrix for the PFW-9 Graded Response Model. Values represent standardized residual correlations (Q3 statistics) between item pairs after model estimation. Positive values indicate local dependence between items beyond the model-implied structure, whereas values close to zero indicate conditional independence. PE = Positive Emotions; EN = Engagement; REL = Relationships; MEAN = Meaning; ACC = Accomplishment; PH = Physical Health; MIND = Mindset; ENV = Environment; ES = Economic Security. | | | | | | | | | |
